# Ethnopharmacological uses of fauna among the people of central Punjab, Pakistan

**DOI:** 10.3389/fvets.2024.1351693

**Published:** 2024-04-12

**Authors:** Abdul Majid Khan, Muhammad Altaf, Tanveer Hussain, M. Haroon Hamed, Umaira Safdar, Amina Ayub, Zaibun-nisa Memon, Adnan Hafiz, Sana Ashraf, Muhammad Shoaib Amjad, Muhammad Majeed, Musheerul Hassan, Rainer W. Bussmann, Arshad Mahmood Abbasi, Mohamed Al-Yafrsi, Hosam O. Elansary, Eman A. Mahmoud

**Affiliations:** ^1^Institute of Zoology, University of the Punjab, Lahore, Pakistan; ^2^Institute of Forest Sciences, The Islamia University of Bahawalpur, Bahawalpur, Pakistan; ^3^Department of Zoology Wildlife and Fisheries, University of Agriculture Faisalabad, Faisalabad, Pakistan; ^4^Department of Zoology, Wildlife and Fisheries, University of Agriculture Faisalabad, Faisalabad, Pakistan; ^5^Department of Zoology, Shah Abdul Latif University, Khairpur, Sindh, Pakistan; ^6^Department of Zoology, University of Sialkot, Sialkot, Punjab, Pakistan; ^7^Department of Zoology, University of Lahore, Sargodha, Pakistan; ^8^Department of Botany, Women University of Azad Jammu and Kashmir Bagh, Bagh, Pakistan; ^9^Department of Botany, University of Gujrat, Gujrat, Punjab, Pakistan; ^10^Department of Ethnobotany, Institute of Botany, Ilia State University, Tbilisi, Georgia; ^11^Alpine Institute of Management and Technology, Dehradun, Uttarakhand, India; ^12^Staatliches Museum Für Naturkunde, Karlsruhe, Germany; ^13^Department of Environment Sciences, COMSATS University Islamabad, Abbottabad, Pakistan; ^14^Department of Plant Production, College of Food & Agriculture Sciences, King Saud University, Riyadh, Saudi Arabia; ^15^Department of Food Science, Faculty of Agriculture, Damietta University, Damietta, Egypt

**Keywords:** Gujranwala, ethnozoology, ethnomedicine, zootherapy, animals, communities

## Abstract

**Introduction:**

The utilization of fauna and fauna-based byproducts in ethnomedicinal usages has been a longstanding human activity, practiced across various cultures worldwide. This study focuses on investigating the utilization of animal-based traditional medicine by the people of Pakistan, specifically in the Gujranwala area.

**Methods:**

Data collection took place from January to September 2019 through interviews with local communities. Ethnomedicinal applications of animal products were analyzed using several indices, including Relative Frequency of Citation (RFC), Relative Popularity Level (RPL), Folk Use Value (FL), and Relative Occurrence Percentage (ROP).

**Results:**

The study identified the use of different body parts of 54 species of animals in treating various diseases and health issues. These include but are not limited to skin infections, sexual problems, pain management (e.g., in the backbone and joints), eyesight issues, immunity enhancement, cold, weakness, burns, smallpox, wounds, poisoning, muscular pain, arthritis, diabetes, fever, epilepsy, allergies, asthma, herpes, ear pain, paralysis, cough, swelling, cancer, bronchitis, girls’ maturity, and stomach-related problems. Certain species of fauna were noted by informers with high “frequency of citation” (FC), ranging from 1 to 77. For instance, the black cobra was the most frequently cited animal for eyesight issues (FC = 77), followed by the domestic rabbit for burn treatment (FC = 67), and the Indus Valley spiny-tailed ground lizard for sexual problems (FC = 66). Passer domesticus and *Gallus gallus* were noted to have the highest ROP value of 99.

**Discussion:**

The findings of this study provide valuable preliminary insights for the conservation of fauna in the Gujranwala region of Punjab, Pakistan. Additionally, screening these animals for medicinally active compounds could potentially lead to the development of novel animal-based medications, contributing to both traditional medicine preservation and modern pharmaceutical advancements.

## 1 Introduction

Zootherapy is described as the use of animal or animal-derived products to treat human ailments (1). The utilization of fauna with therapeutic characteristics is still a popular practice across the world (2). Zootherapy methods and materials are used in both folk and modern medicine to treat different kinds of sicknesses (3–7). It has been found that over 13% of the medications used in traditional Chinese medicine are derived from fauna. In Ayurvedic medicine, faunal products make up 15–20% of the medications. More than 111 medications in Tibetan medicine contain fauna-based components (8–10).

Many communities are rapidly losing ethnomedicinal expertise, making it increasingly necessary to capture this information before it is lost (1, 11–17). The utilization of fauna and fauna-based yield in folk therapy has been under-documented, most likely due to the dominance of plants in folk medical systems (18, 19). Pakistan has a rich fauna diversity, including 668 species of birds (20), 195 species of mammals (21), 24 species of amphibians (22), and 195 species of reptiles (23).

Gujranwala has a diverse range of fauna and biodiversity. This region, with its plains and different ecosystems, is home to an amazing variety of wildlife. Dominant avian fauna is documented in Gujranwala, i.e., *Acridothere ginginianus*, *Acridothere tristis*, *Apus affinis*, *Athene brama*, *Bubulcus ibis*, *Cercomela fusca*, *Columba livia*, *Corvus splendens*, *Egretta garzetta*, *Hirundo rustica*, *Hoplopterus indicus*, *Milvus migrans migrans*, *Nectarinia asiatica*, and *Passer domesticus* (24), while important mammalian fauna of the area is reported as, i.e., *Suncus etruscus*, *Funnambulus pennantii*, *Rattus rattus*, *Mus musculus*, *Herpestes javanicus*, and *Herpestes edwardsi* (25–27), and more than 30 species of freshwater are documented along Gujranwala (28), and prominent herptiles are *Saara hardwickii*, Var*anus bengalensis*, *Duttaphrynus stomaticus*, *Hemidactylus flaviviridis*, *Hoplobatrachus tigerinus*, *Aspideretes gangeticus*, *Lissemys punctate*, *Calotes versicolor*, and *Eryx johnii* (29).

Knowing the conservation and management of biocultural systems requires ethnozoological study. Traditional usages of fauna species, e.g., food (30–37), medicine (32, 38–42), trade (43), etc., can endorse attitude that aids in the conservation of these animals; however, if they are practiced in an unsustainable manner or are influenced by economic and political factors, they may have a negative impact on or even endanger these animals. Local populations’ usage of animal species in traditional medicine and for cultural purposes must be evaluated in connection to other issues such as climate and habitat changes (44, 45). There is a global need to identify innovative techniques to cope with the current catastrophe of loss of biodiversity (46), and ethnozoology gives crucial insights into local community practices, allowing conservation efforts to successfully collaborate with resource managers to enhance the overall veracity of biological structures (47, 48). A number of studies have been conducted to date that has documented the use of animal parts in traditional medicine in various areas of Pakistan (31, 32, 49–61); however, ethnomedicinal applications of animals in Gujranwala have never been described. This research on the medical applications of fauna by the people of Gujranwala district in Pakistan is part of a larger plan to record the usage of fauna by local populations across Pakistan (18, 29, 62–64). The research on the ethnopharmacological uses of fauna among the inhabitants of central Punjab, Pakistan, aims to investigate and describe traditional understandings and methods of using local animal resources for medicinal reasons in this area. By conducting an in-depth inquiry into the fauna-based remedies used by communities of indigenous peoples, the study hopes to contribute to the preservation of traditional healing practices, shed light on the possible medicinal qualities of these animals, and provide insights for the conservation of biodiversity.

## 2 Materials and methods

### 2.1 Study site and climate

**Figure 1:**
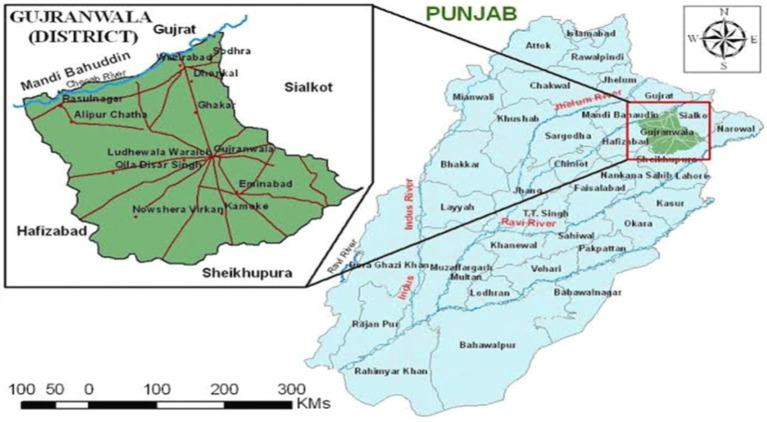
Study site map.

**Figure 2:**
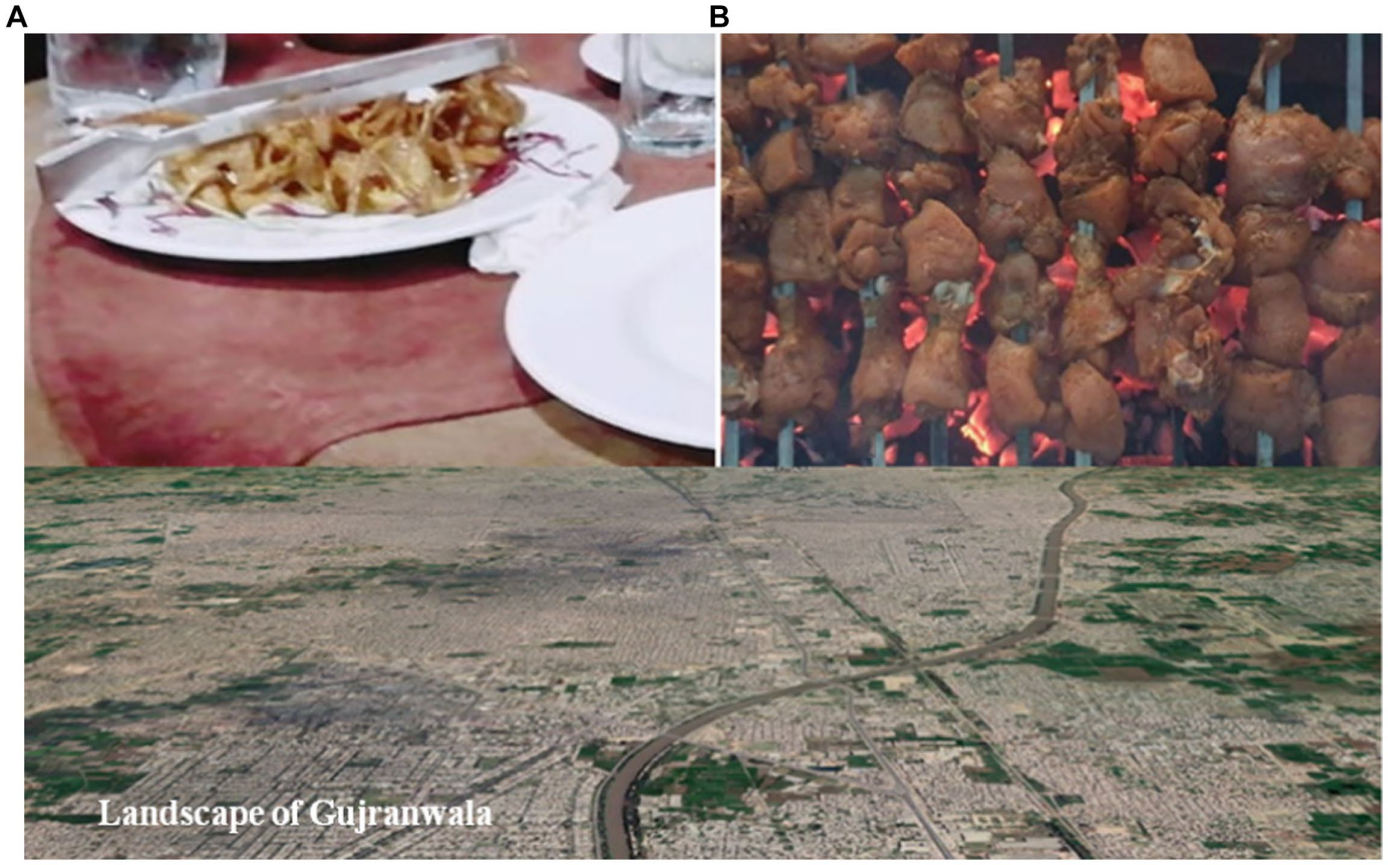
**(A,B)** Famous meat (**A**: quail meat, **B**: chicken) and landscape of Gujranwala.

Gujranwala is the city and capital of Gujranwala Division, Punjab, Pakistan. It is also known as the “City of Wrestlers.” Gujranwala is Pakistan’s fifth-most populous city. Founded in the eighteenth century, Gujranwala is a relatively modern town compared to other nearby old cities in northern Punjab (Figure 1). The people of the area like to eat meat. The coordinates of Gujranwala are 32°9′24″N 74°11′24″E. Gujranwala has a hot, semi-arid climate. During the summer (June to September), the temperature reaches 40 °C. The coldest months are typically November through February, when temperatures can dip to an average of 5°C. During the other months, the average rainfall is approximately 25 mm. There is very little rain from October to May (Figures 2A,B).

### 2.2 Data collection

Semi-structured interviews and questionnaires were collected from 100 respondents (i.e., traditional health practitioners, farmers, teachers, hunters, and herdsmen). We selected random respondents from the community who have knowledge about traditional therapy. Respondents were chosen based on their ethnomedicinal and ethnocultural recognition of the customary remedial and societal value of herptiles, fish, animals, and fowl. Birds were identified using field guides, “The Birds of Pakistan.” Data were statistically analyzed using five indices, including (i) the “fidelity level” (FL), (ii) the “relative popularity level” (RPL), and (iii) “rank order priority.”

### 2.3 The Fidelity level

FL was analyzed with the help of a formula as follows (65):


$$FL\;\left(\%\right)=N_{p}/FC\times 100.$$

where “N_p_” = number of respondents with vital chronic diseases for certain breeds of animals; FC = frequency of citation for ethnocultural utilization of specific creatures.

### 2.4 The relative popularity level

RPL was described previously by Friedman et al. (66); creatures were separated into two gatherings, for example, (i) “famous” and (ii) “disagreeable.” (i) Popular creatures are those species that were expressed for the greater part of the most extreme recurrence of reference (FC). (ii) The left-over creatures were recorded as disagreeable. While for famous (creature species) an even line was non-existent specifically, the normal numeral of employments per creature is free of the recurrence of reference (FC), who perceives the creatures; along these lines, the normal numeral of employments of mainstream creature animal varieties does not improve with the addition to recurrence of reference, who refers to the creature species for any clinical use. For the mainstream creature species, the RPL was set at 1. For creature species in disliked gatherings, the overall notoriety level is under 1.0.

### 2.5 Rank order priority

ROP is utilized to group the fauna species (66, 67) and was examined using the following formula:


ROP=FL×RPL

## 3 Results and discussion

### 3.1 Informant selection for ethnozoological data collection

**Figure 3:**
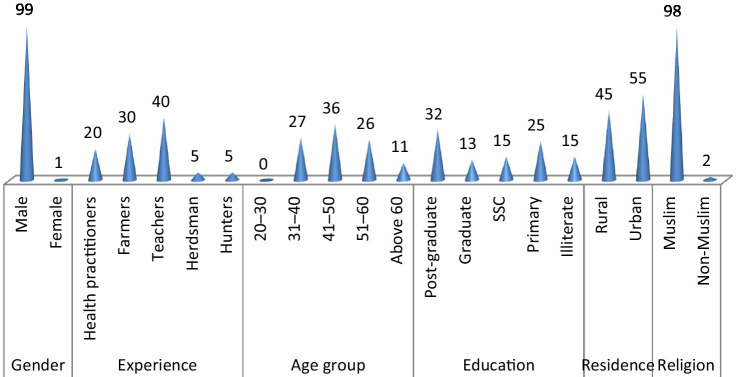
Selected informants on the ethnozoological usage of the documented species.

During the years 2018–2020, ethnozoological information was gathered; prior to the data collection, a reconnaissance survey was conducted to ensure accessibility across the study area. Using the snowball technique, we collected the traditional endemic knowledge using semi-structured questionnaires followed by group discussions. The informants selected were mostly from urban (55%). Furthermore, the selected informants were classified into different professions and age groups. The maximum number of informants were educated (92.9%). Upon data collection, men showed ascendency over women due to cultural and religious limitations. Women are not allowed to talk with strangers due to religious and social norms, and they are usually involved in house chores. The complete details can be found in Figure 3. It is documented that older people have more insights about traditional knowledge as compared with younger people, and it is also noted that uneducated and less educated people have more reliance on traditional knowledge as compared with highly educated people.

### 3.2 Taxonomy and ethnozoological inventory

In the ethno-scientific domain, we documented a total of 54 species used in Gujranwala, Pakistan, classified into different categories, i.e., herptiles (*n* = 7), fish (*n* = 16), mammals (*n* = 13), birds (*n* = 15), and invertebrates (*n* = 3). The complete inventory is provided in Table 1. The maximum usage of the fish species in the region can be attributed to the presence of the two important rivers (Ravi and Chenab) inhabiting varieties of fish fauna; also, the locals have a potential traditional knowledge of fish utilization.

**Table 1:** A detailed analysis of the ethnozoology of Gujranwala, Punjab.

No.	Common name Scientific name (species authority) Punjabi name	Uses of body parts	Mode of use	Diseases	FC	RFC	Np	RPL	FL	ROP
Herptiles										
1	Indus Valley toad *Bufo stomaticus* (Lutkin, 1862) Ghariallo daddo	S	T	Skin diseases	8.0	0.08	2	0.23	25.00	6
2	Indian Bullfrog *Hoplobatrachus tigerinus* (Daudin, 1802) Wada daddo	F	T	Sexual problems	13.0	0.13	3	0.37	23.08	8
				Pain in backbone	28.0	0.28	6	0.79	21.43	17
				Pain in joints	12.0	0.12	4	0.34	33.33	11
3	Indus Valley spiny-tail ground lizard *Uromastyx hardwickii* (Strauch, 1863) Sanda	F	T	Pain in joints	4.0	0.04	1	0.11	25.00	3
				Pain in backbone	23.0	0.23	8	0.65	34.78	23
				Sexual problems	66.0	0.66	28	1.00	42.42	42
4	Bengal Monitor *Varanus bengalensis* (Daudin, 1802) Goh	F	T	Pain in backbone	2.0	0.02	1	0.06	50.00	3
				Sexual problems	2.0	0.02	1	0.06	50.00	3
5	Rock python *Python molurus* (Linnaeus, 1758) Azdha sap	F	T	Pain in joints	2.0	0.02	1	0.06	50.00	3
				Sexual problems	11.0	0.11	7	0.31	63.64	20
6	Black cobra *Naja naja naja* (Linnaeus, 1768) Kala naag	S, F	T	Sexual problems	19.0	0.19	3	0.54	15.79	8
				Eyesight	77.0	0.77	13	1.00	16.88	17
7	Russell’s chain Viper *Daboia russelii russelii* (Shaw and Nodder, 1797) Koriwala	F	T	Sexual problems	9.0	0.09	2	0.25	22.22	6
				Pain in joints	7.0	0.07	2	0.20	28.57	6
Fishes										
8	Grass carp *Ctenopharyngodon idella* (Valenciennes, 1844) Grass carp	B, M, F	T, O	Eye problems	7.0	0.07	2	0.20	28.57	6
				Blindness in night	6.0	0.06	2	0.17	33.33	6
				Enhance memory	10.0	0.1	2	0.28	20.00	6
				Sexual problems	19.0	0.19	3	0.54	15.79	8
9	Common carp *Cyprinus carpio* (Linnaeus, 1758) Gulfam	B, M, F	T, O	Eye problems	8.0	0.08	2	0.23	25.00	6
				Blindness in night	3.0	0.03	2	0.08	66.67	6
				Enhance memory	13.0	0.13	3	0.37	23.08	8
				Sexual problems	10.0	0.1	2	0.28	20.00	6
				Enhance immunity	21.0	0.21	2	0.59	9.52	6
10	Silver carp *Hypophthalmichthys molitrix* (Valenciennes, 1844) Silver carp	B, M, F	T, O	Eye problems	8.0	0.08	3	0.23	37.50	8
				Blindness in night	3.0	0.03	1	0.08	33.33	3
				Enhance memory	23.0	0.23	2	0.65	8.70	6
				Sexual problems	28.0	0.28	4	0.79	14.29	11
				Enhance immunity	7.0	0.07	1	0.20	14.29	3
11	Mrigal carp *Cirrhinus mrigala* (Hamilton, 1822) Mori	B, M, F	T, O	Eye problems	5.0	0.05	1	0.14	20.00	3
				Blindness in night	8.0	0.08	1	0.23	12.50	3
				Enhance immunity	12.0	0.12	3	0.34	25.00	8
				Cold	10.0	0.1	3	0.28	30.00	8
				Sexual problems	7.0	0.07	2	0.20	28.57	6
12	Reba carp *Cirrhinus reba* (Hamilton, 1822) Reba Machhali	B, M, F	T, O	Eye problems	1.0	0.01	1	0.03	100.00	3
				Blindness in night	4.0	0.04	1	0.11	25.00	3
				Enhance memory	5.0	0.05	1	0.14	20.00	3
				Sexual problems	3.0	0.03	1	0.08	33.33	3
				Enhance immunity	5.0	0.05	1	0.14	20.00	3
13	Raho *Labeo rohita* (Hamilton, 1822) Raho	B, M, F	O	Pain in joints	8.0	0.08	2	0.23	25.00	6
				Sexual problems	36.0	0.36	18	1.00	50.00	50
				Eyesight	12.0	0.12	4	0.34	33.33	11
				Enhance memory	14.0	0.14	3	0.39	21.43	8
				Enhance immunity	12.0	0.12	2	0.34	16.67	6
14	Orangefin labeo *Labeo calbasu* (Hamilton, 1822) Kalbans	B, M, F	T, O	Enhance memory	1.0	0.01	1	0.03	100.00	3
				Pain in body	2.0	0.02	1	0.06	50.00	3
				Sexual problems	12.0	0.12	7	0.34	58.33	20
				Enhance immunity	10.0	0.1	3	0.28	30.00	8
15	Catla *Gibelion catla* (Hamilton, 1822) Thaila	B, M, F	O	Enhance memory	4.0	0.04	2	0.11	50.00	6
				Sexual problems	22.0	0.22	3	0.62	13.64	8
16	Spotted snakehead *Channa punctata* (Bloch, 1793) Dola	B, M, F	O	Enhance memory	5.0	0.05	3	0.14	60.00	8
				Enhance immunity	30.0	0.3	20	0.85	66.67	56
17	Great snakehead *Channa marulius* (Hamilton, 1822) Soul	B, M, F	O	Sexual problems	19.0	0.19	11	0.54	57.89	31
				Enhance potential	12.0	0.12	8	0.34	66.67	23
18	Nile tilapia *Oreochromis niloticu* (Linnaeus, 1758) Tilapia	B, M, F	T	Enhance immunity	25.0	0.25	12	0.70	48.00	34
				Burn	4.0	0.04	2	0.11	50.00	6
19	Rita *Rita rita* (Hamilton, 1822) Khaga	B, M, F	O, T	Enhance immunity	13.0	0.13	7	0.37	53.85	20
				Sexual problems	7.0	0.07	4	0.20	57.14	11
20	Goonch *Bagarius bagarius* (Hamilton, 1822) Foji Khaga	B, M, F	O	Enhance immunity	21.0	0.21	9	0.59	42.86	25
				Sexual problems	25.0	0.25	9	0.70	36.00	25
21	Gangetic mystus *Mystus cavasius* (Hamilton, 1822) Tangra Machhali	B, M, F	O	Small pox	2.0	0.02	1	0.06	50.00	3
				Enhance immunity	16.0	0.16	2	0.45	12.50	6
22	Zig-zag eel *Mastacembelus armatus* (Lacepède, 1800) Baam Machhali	B, M, F	O	Sexual problems	7.0	0.07	2	0.20	28.57	6
				Enhance immunity	16.0	0.16	3	0.45	18.75	8
23	Wallago catfish *Wallago attu* (Bloch & Schneider, 1801) Mali	B, M, F	O	Enhance memory	5.0	0.05	2	0.14	40.00	6
				Enhance immunity	11.0	0.11	4	0.31	36.36	11
Mammals										
24	Cow *Bos gaurus* (C. H. Smith, 1827) Gay	B, M, F	O, T	Wounds in feet	12.0	0.12	7	0.34	58.33	20
				Pain in body	21.0	0.21	19	0.59	90.48	54
				Engulf of poisonous things	12.0	0.12	2	0.34	16.67	6
25	Buffalo *Bubalus bubalis* (Linnaeus, 1758) Mujh	B, M, F	O, T	Wounds in feet	12.0	0.12	8	0.34	66.67	23
				Pain in body	37.0	0.37	21	1.00	56.76	57
				Engulf of poisonous things	7.0	0.07	4	0.20	57.14	11
26	Camel *Camelus dromedaries* (Linnaeus, 1758) Ount	MI, BL, M	Oral Topical	Pain in muscles	2.0	0.02	1	0.06	50.00	3
				Pain in joints	2.0	0.02	1	0.06	50.00	3
				Diabetes	37.0	0.37	22	1.00	59.46	59
27	Goat *Capra aegagrus hircus* (Linnaeus, 1758) Bakri	MI	Oral	Sexual problems	41.0	0.41	22	1.00	53.66	54
				Cold	2.0	0.02	1	0.06	50.00	3
				Fever	2.0	0.02	1	0.06	50.00	3
28	Northern palm squirrel *Funnambulus pennanti* (Wroughton, 1905) Gulahri	M, H	O, T	Epilepsy	7.0	0.07	2	0.20	28.57	6
				Skin diseases	7.0	0.07	2	0.20	28.57	6
				Allergy	7.0	0.07	2	0.20	28.57	6
29	Small Indian mongoose *Herpestes javanicus* (Geoffroy Saint-Hilarie, 1818) Neola	F	T	Sexual problems	6.0	0.06	2	0.17	33.33	6
				Pain in joints	6.0	0.06	2	0.17	33.33	6
				Pain in backbone	6.0	0.06	2	0.17	33.33	6
30	Humans *Homo sapiens* (Linnaeus, 1758) Adam	SA	T	Herpes	27.0	0.27	5	0.76	18.52	14
				Ear pain	27.0	0.27	3	0.76	11.11	8
				Eye problems	27.0	0.27	5	0.76	18.52	14
31	Desert hare *Lepus nigricollis dayanus* (F. Cuvier, 1823) Jungli khargush	M, L, BL	O,T	Asthma	12.0	0.12	4	0.34	33.33	11
				Burn	12.0	0.12	4	0.34	33.33	11
				Face paralysis	12.0	0.12	4	0.34	33.33	11
32	Indian Pangolin *Manis crassicaudata* (É.Geoffroy Saint-Hilaire, 1803) Pangolin, Sipple	SC, M	T	Feet swelling	11.0	0.11	4	0.31	36.36	11
				Sexual problems	2.0	0.02	1	0.06	50.00	3
				Cancer	2.0	0.02	1	0.06	50.00	3
33	Domestic rabbit *Oryctolagus cuniculus* (Linnaeus, 1758) Khargush, Saya	T, BL	T	Burn	67.0	0.67	34	1.00	50.75	51
				Enhance potential	2.0	0.02	1	0.06	50.00	3
				Face paralysis	2.0	0.02	1	0.06	50.00	3
34	Sheep *Ovis aries* (Linnaeus, 1758) Bairh	F, MI, M	O,T	Burn	26.0	0.26	22	0.73	84.62	62
				Enhance potential	26.0	0.26	22	0.73	84.62	62
				Pain in joints	26.0	0.26	22	0.73	84.62	62
35	Indian flying fox bat *Pteropus giganteus* (Brünnich, 1782) Chamga-dar	F	T	Pain in body	2.0	0.02	1	0.06	50.00	3
				Pain in joints	2.0	0.02	1	0.06	50.00	3
				Pain in backbone	2.0	0.02	1	0.06	50.00	3
36	Bear *Ursus thibetanus* (Cuvier, 1823) Richh	F	T	Sexual problems	3.0	0.03	2	0.08	66.67	6
				Pain in joints	3.0	0.03	2	0.08	66.67	6
				Pain in backbone	3.0	0.03	2	0.08	66.67	6
Birds										
37	Common Myna *Acridotheres tristis* (Linnaeus, 1766) Lali	M	O	Cough	9.0	0.09	2	0.25	22.22	6
				Fever	9.0	0.09	2	0.25	22.22	6
38	Domestic Duck *Anas platyrhynchos domesticus* (Linnaeus, 1758) Batakh	E	O	Eye problems	9.0	0.09	2	0.25	22.22	6
				Cold	9.0	0.09	2	0.25	22.22	6
39	Mallard *Anas platyrhynchos* (Linnaeus, 1758) Nilsir	M, E	O	Face paralysis	4.0	0.04	3	0.11	75.00	8
				Cold	4.0	0.04	3	0.11	75.00	8
40	Tawny Eagle *Aquila rapax* (Temminck, 1828) Chhota baaz	F	T	Skin diseases	3.0	0.03	2	0.08	66.67	6
				Cancer	3.0	0.03	2	0.08	66.67	6
41	Spotted Little Owlet *Athene brama* (Temminck, 1821) Ullo	BL	T	Sexual problems	10.0	0.1	2	0.28	20.00	6
				Skin diseases	10.0	0.1	2	0.28	20.00	6
42	Blue Rock Pigeon *Columba livia* (F.Gmelin, 1789) Jangli kabotar	M, FE	O	Face paralysis	44.0	0.44	22	1.00	50.00	50
				Cold	44.0	0.44	22	1.00	50.00	50
43	Common Quail *Coturnix coturnix* (Linnaeus, 1758) Batera	M	O	Enhance memory	10.0	0.1	7	0.28	70.00	20
				Sexual problems	10.0	0.1	7	0.28	70.00	20
44	Black partridge *Francolinus francolinus* (Linnaeus, 1766) Kala tittar	M	O	Bronchitis	19.0	0.19	11	0.54	57.89	31
				Enhance potential	19.0	0.19	11	0.54	57.89	31
45	Domestic Chicken *Gallus gallus* (Linnaeus, 1758) Murghi	E, M	O	Fever	35.0	0.35	35	0.99	100.00	99
				Cold	35.0	0.35	35	0.99	100.00	99
46	Bonnelli’s Eagle *Hieraaetus fasciatus* (Sibley & Monroe, 1990) Baaz	F	T	Cancer	3.0	0.03	1	0.08	33.33	3
				Skin diseases	3.0	0.03	1	0.08	33.33	3
47	House Sparrow *Passer domesticus* (Linnaeus, 1758) Chiri	M	O	Enhance potential	35.0	0.35	35	0.99	100.00	99
				Face paralysis	35.0	0.35	35	0.99	100.00	99
48	Indian Ring Dove *Streptopelia decaocto* (Frivaldszky, 1838) Kogi	M	O	Enhance maturity	5.0	0.05	2	0.14	40.00	6
				Cold	5.0	0.05	2	0.14	40.00	6
49	Oriental turtle Dove *Streptopelia orientalis* (Latham, 1790) Kogi	M	O	Enhance maturity	5.0	0.05	2	0.14	40.00	6
				Cold	5.0	0.05	2	0.14	40.00	6
50	Little Brown Dove *Streptopelia senegalensis* (Linnaeus, 1766) Chhoti kogi	M	O	Enhance maturity	5.0	0.05	2	0.14	40.00	6
				Cold	5.0	0.05	2	0.14	40.00	6
51	Red Turtle Dove *Streptopelia tranquebarica* (Hermann, 1804) Lal kogi	M	O	Enhance maturity	5.0	0.05	2	0.14	40.00	6
				Cold	5.0	0.05	2	0.14	40.00	6
Invertebrates										
52	European honey bee *Apis mellifera* (Linnaeus, 1758) Shahd Makhi	HO	O	Stomach	24.0	0.24	11	0.68	45.83	31
				Eye diseases	24.0	0.24	11	0.68	45.83	31
				Skin diseases	24.0	0.24	11	0.68	45.83	31
				Diabetics	24.0	0.24	11	0.68	45.83	31
53	Earthworm *Pheretima hawayana* (Rosa, 1891) Gandoya	WB	O	Pain in backbone	12.0	0.12	9	0.34	75.00	25
54	Common beak *Libythea lepita* (Moore, 1857) Titli	WB	O	Antibacterial	41.0	0.41	4	1.00	9.76	10

O, oral; T, topical; S, skin; SA, saliva; SC, scale; M, meat; MI, milk; F, fat; E, egg; WB, whole body; HO, honey; H, hair; FE, feather; B, brain; BL, blood; T, tail; L, liver.

**Figure 4:**
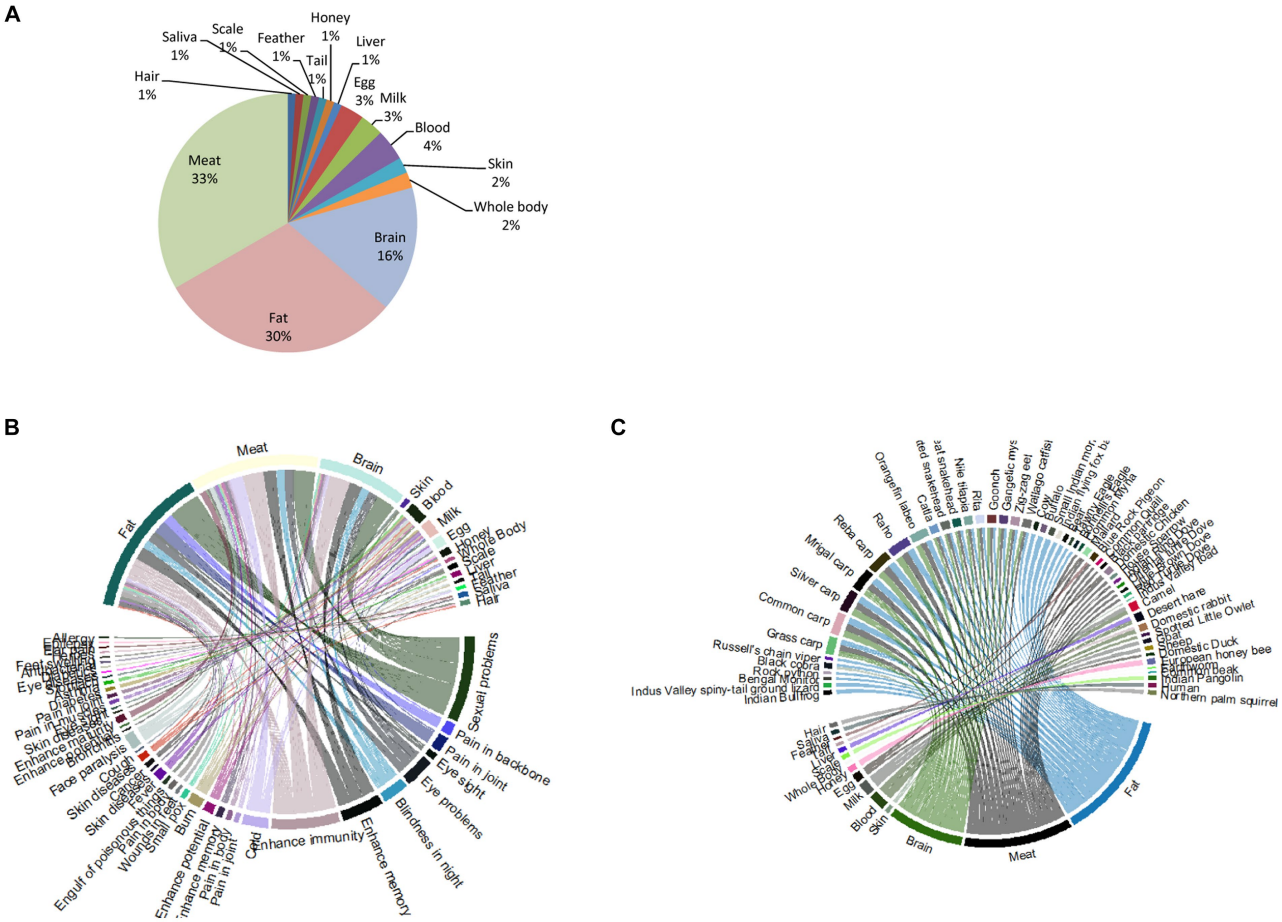
**(A)** Percentage of different body parts of fauna used to treat a variety of diseases in the study area. **(B)** The body parts are used to treat diseases. **(C)** Ethnopharmacological uses of body parts of animal species in the study area.

Upon interpreting the results, it was revealed that different body parts of the documented species were employed against a variety of diseases. The most frequently used parts were meat (33%), followed by fat (30%), and brain (16%; Figures 4A–C).

The fat of animals is the second most consumed body part of animals. The fat of 31 species of animals, i.e., Indian Bullfrog, Indus Valley spiny-tail ground lizard, Bengal monitor, rock python, black cobra, Russell’s chain viper, grass carp, common carp, silver carp, mrigal carp, Reba carp, raho, Orangefin labeo, catla, spotted snakehead, great snakehead, Nile tilapia, Rita, goonch, Gangetic mystus, zig–zag eel, wallago catfish, cow, buffalo, small Indian mongoose, Indian flying fox bat, bear, tawny eagle, and Bonelli’s eagle, is used to treat wounds in feet, small pox, skin diseases, sexual problems, pain in joints, pain in body, pain in backbone, fever, eyesight and eye problems, enhance potential, memory, and immunity, engulf of poisonous things, cold, cancer, burn, and blindness in the night (Figures 4A,B).

Similarly, the brain of animal species, i.e., grass carp, common carp, silver carp, mrigal carp, Reba carp, raho, Orangefin labeo, catla, spotted snakehead, great snakehead, Nile tilapia, Rita, goonch, Gangetic mystus, zig–zag eel, and wallago catfish, is used to treat small pox, sexual problems, pain in joints, pain in the body, eyesight and eye problems, enhance potential, memory, and immunity, cold, burn, and blindness at night (Figures 4A,B).

Similarly, the skin of animal species, i.e., the Indus Valley toad and black cobra catfish, is used to cure skin diseases, sexual problems, and eyesight, while the blood of camel, desert hare, domestic rabbit, and spotted little owlet is used to cure pain in muscles, pain in joints, diabetes, asthma, burns, face paralysis, burn, enhance potential, sexual problems, and skin diseases. However, the milk of camel, goat, and sheep is used to treat pain in muscles, pain in joints, diabetes, sexual problems, cold, fever, burn, and enhance potential (Figures 4A,B).

### 3.3 Ethnomedicinal use of herptile and fish

**Figure 5:**
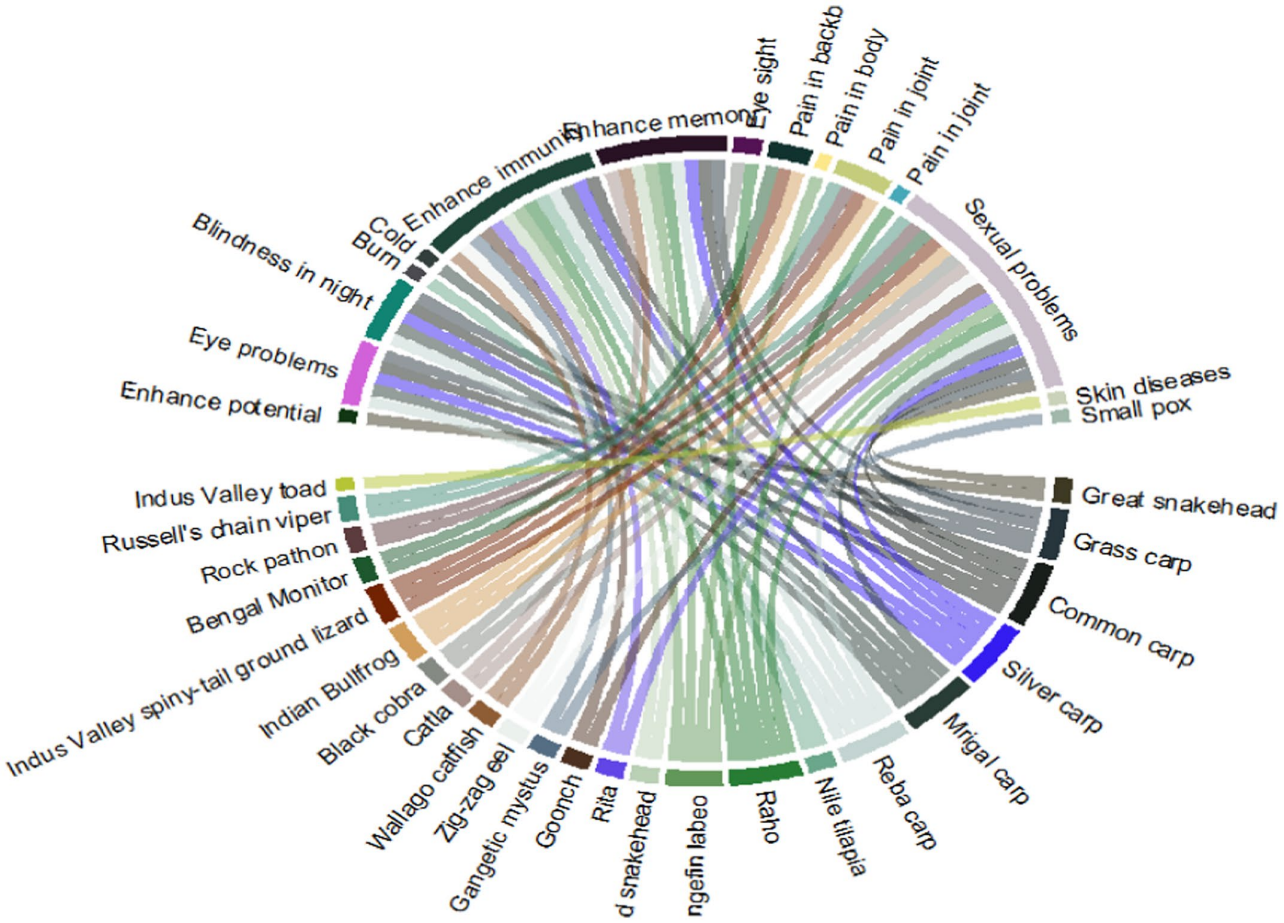
Herptiles and fish species used to cure different kinds of sicknesses in the study area.

In the present study, we found that the local people use the herptiles and fish for different ailments. Sexual problems were found to be treated by most of the species, followed by “enhanced immunity” (Figure 5).

### 3.4 Ethnomedicinal use of mammals

**Figure 6:**
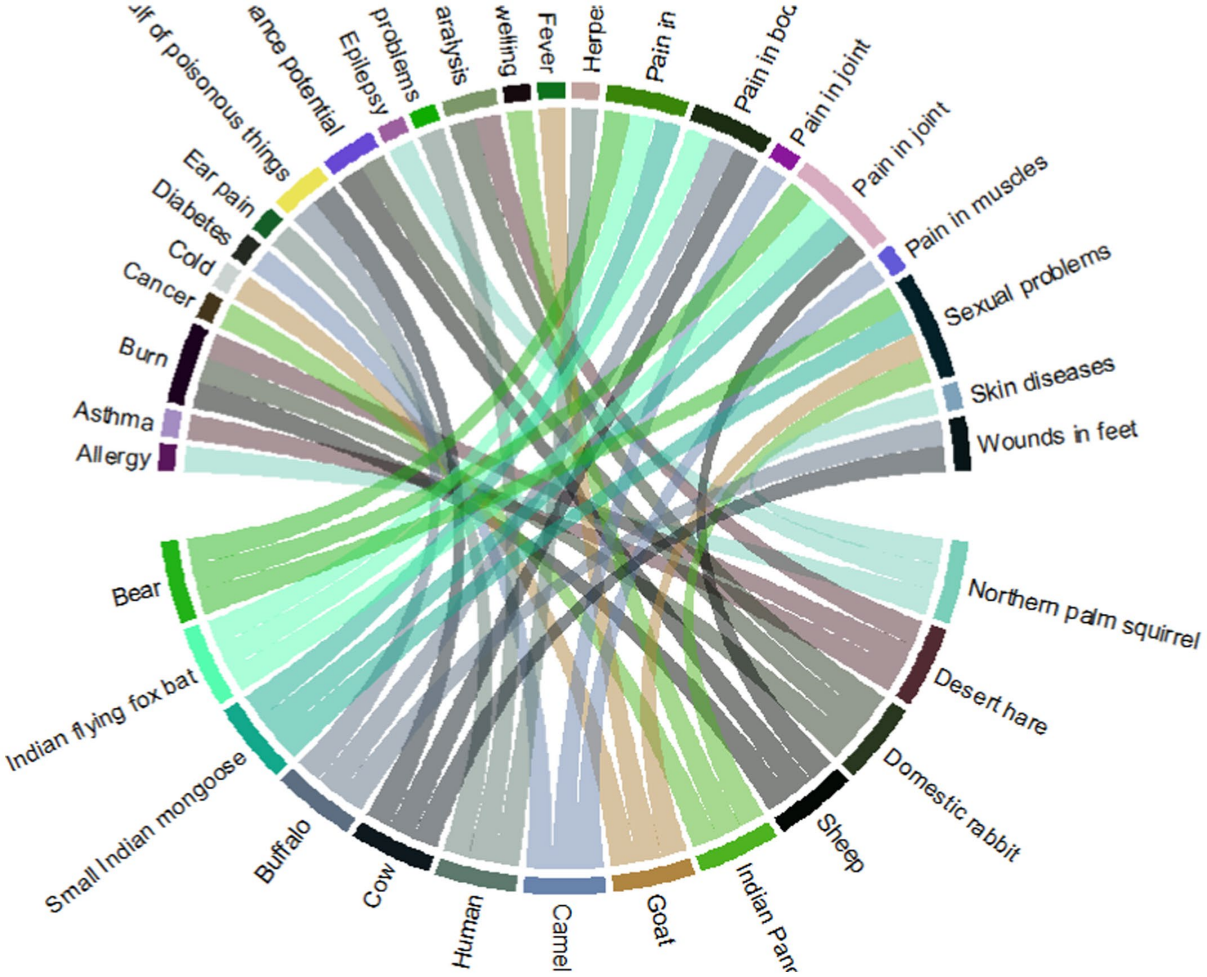
Mammalian species used to cure different kinds of sicknesses in the study area.

The present study revealed that 23 diseases were treated by the documented mammals (Figure 6). The maximum use of the species to treat cold is due to the belief that the meat has the potential to overcome cough and cold. Also, meat is rich in protein, which in turn provides body strength.

### 3.5 Ethnomedicinal use of aves and invertebrates

**Figure 7:**
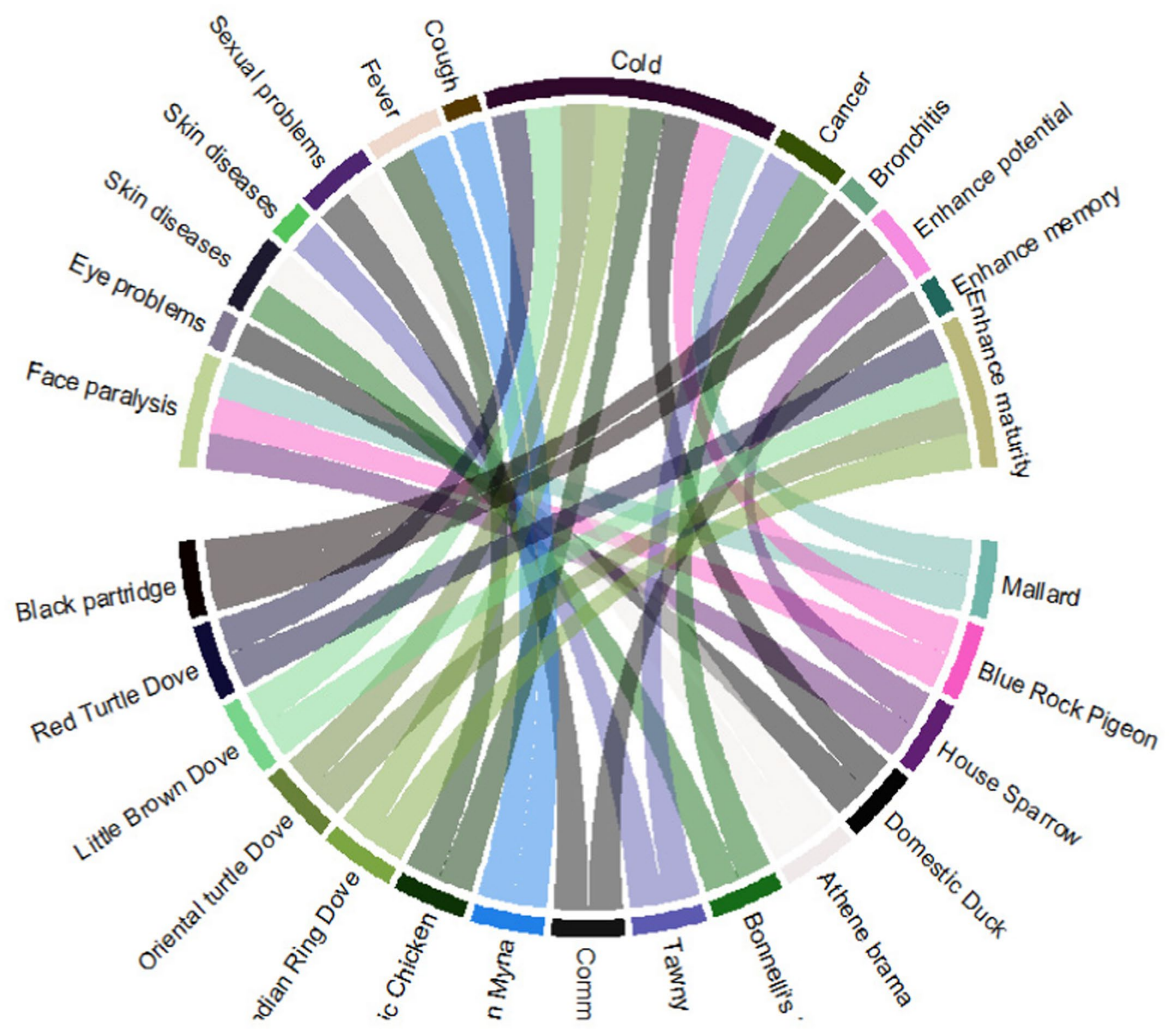
Avian species used to cure different kinds of sicknesses in the study area.

**Figure 8:**
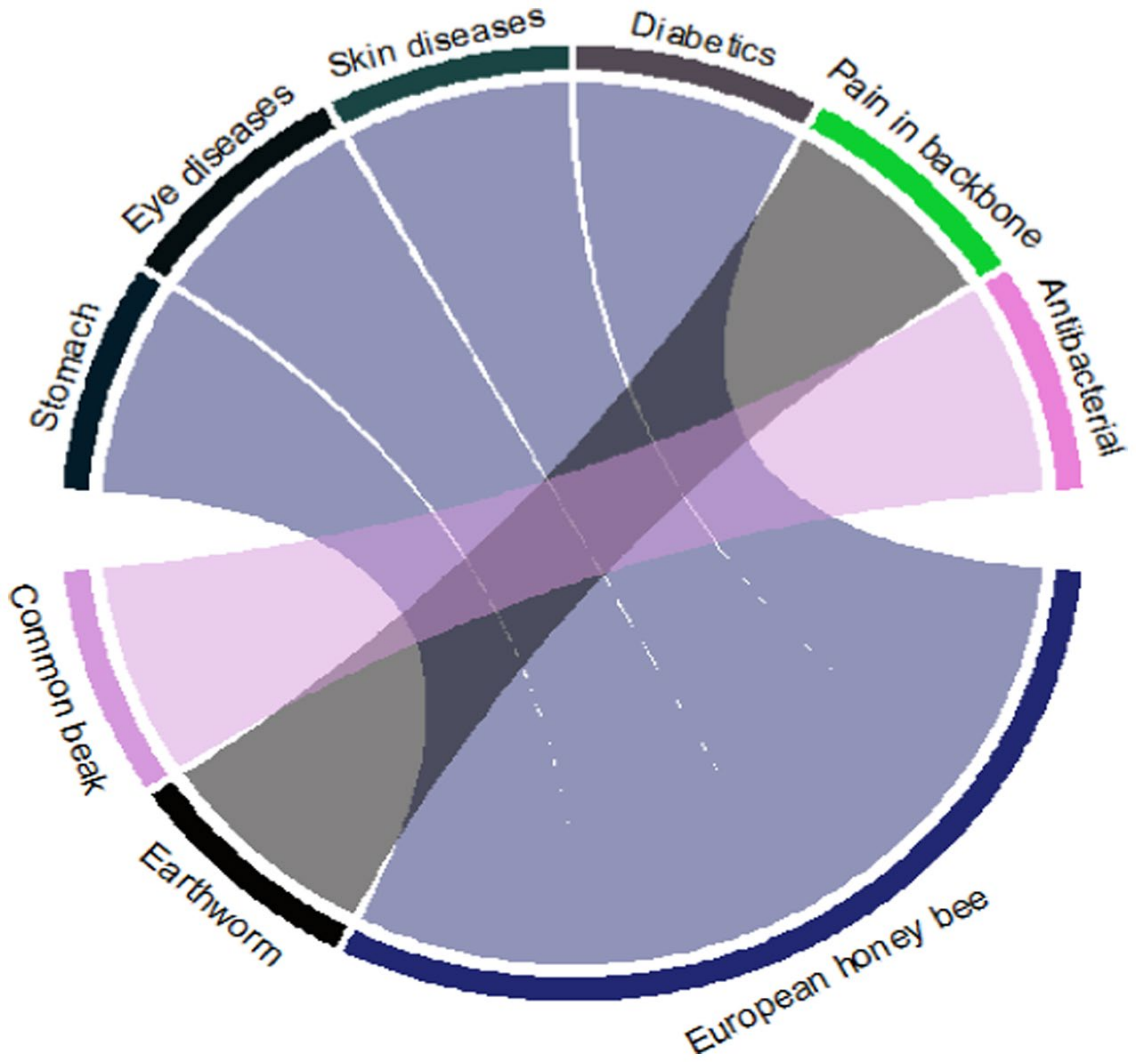
Invertebrate species used to cure different kinds of sicknesses in the study area.

In the present study, 15 birds were reported to treat 13 diseases. The most frequent diseases treated were “cold” followed by “enhanced maturity” and “face paralysis” (Figure 7). Only three invertebrate species were documented, i.e., European honey bee, earthworm, and common beak, to treat different diseases. The European honey bee was recorded for ascendancy as the said species treated a maximum number of diseases (stomach, eye diseases, skin diseases, and diabetics), contrary to the other two invertebrates (Figure 8).

### 3.6 Frequency of citation

**Figure 9:**
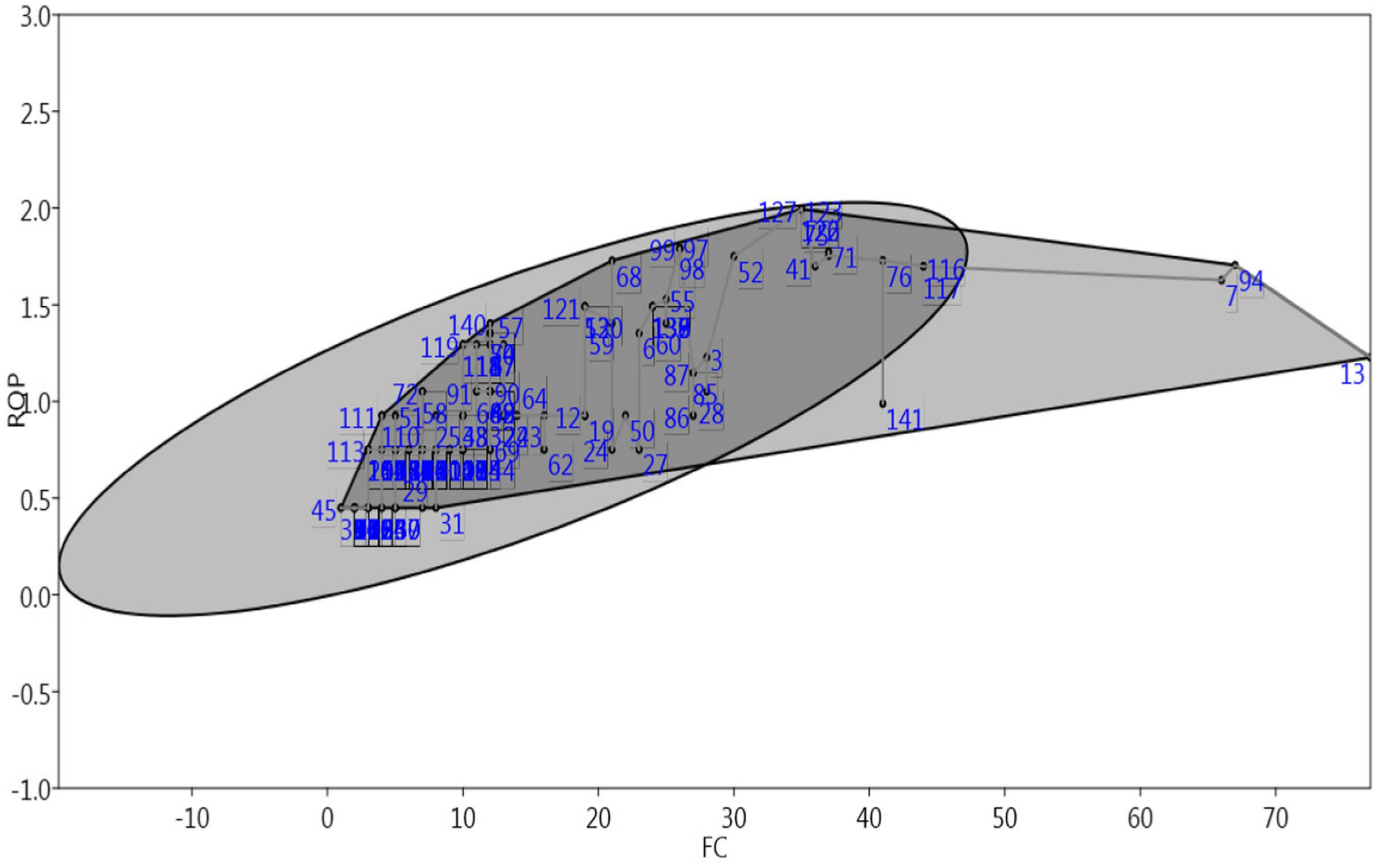
A comparison of FC and ROP values.

In the present study, different species were reported by a different number of informants. The FC ranged between 1 and 77 (Table 1; Figure 9). The highest value of FC (77) was obtained for Black cobra (for eyesight), followed by domestic rabbit (for burn) with FC = 67, and Indus Valley spiny-tail ground lizard (for sexual problems; FC = 66). The lowest value of FC = 1 was recorded for the Orangefin labeo (to enhance memory) and Reba carp (for eye problems).

### 3.7 The Fidelity level

**Figure 10:**
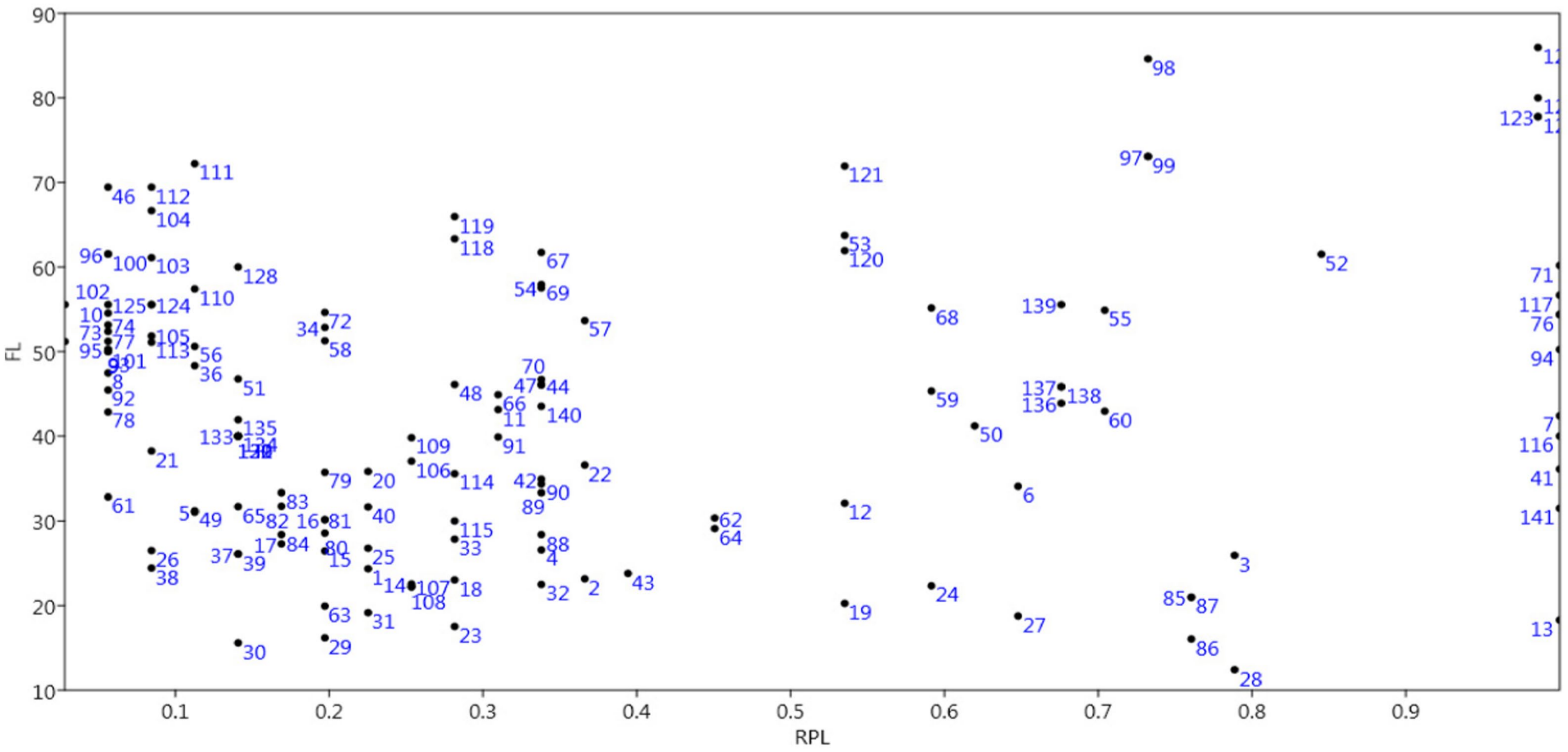
A comparison of FL and RPL values.

According to Altaf et al. (18), FL is used for the identification of the species that are most preferred in a region by the local inhabitants for curing different diseases. Any species with the maximum medicinal uses in a region is known to have the highest fidelity level (54). A complete list of the fidelity levels of the documented species is provided in Table 1. The highest FL (100; Figure 10) was registered for Orangefin labeo, *Labeo calbus* (for enhanced memory); Reba carp, *Cirrhinus reba* (for eye problems); domestic chicken, *Gallus* (for fever and cold); house sparrow (to enhance potential and face paralysis); followed by cows (90.48) to treat pain in the body and *Ovis aries* (84.62) to treat burns, enhance potential, and to treat pain in joints. The lowest FL (8.70) was recorded for *Hypophthalmichthys molitrix* (to enhance memory).

### 3.8 The relative popularity level

The relative popularity level (RPL) of the species can be seen in Table 1. We grouped the species into two categories (popular and unpopular; Figure 9). Species with RPL 1 are considered to be popular, and these include species, such as Indus Valley spiny-tail ground lizard, *Uromastyx hardwickii;* Black cobra, *Naja naja naja;* Raho, *Labeo rohita;* Buffalo, *Bubalus bubalis;* Camel, *Camelus dromedaries;* Goat, *Capra aegagrus hircus;* Domestic rabbit, *Oryctolagus cuniculus;* Blue Rock Pigeon, *Columba livia;* and Common beak, *Libythea lepita*.

### 3.9 Rank order priority

Rank order priority (ROP) is employed to assign an appropriate grade to the documented species with different FL values. The calculated ROP values for each species are presented in Table 1. *Passer domesticus* and *Gallus gallus* were the species with the highest ROP (99), followed by *Ovis aries* (62), *Camelus dromedaries* (59), *Bubalus bubalis* (57), *Channa punctata* (56), *Labeo rohita* (50), and *Uromastyx hardwickii* (42; Table 1; Figure 7).

## 4 Discussion

Meat contains nitrogenous and non-nitrogenous substances, water, lipids, sodium, magnesium, glycogen, lactic acid, potassium, iron, calcium, phosphorus, and chlorine (68, 69). Meat composition varies owing to the effects of many environmental elements and internal characteristics such as animal species, diet, muscle, breed, and sex (70). Poultry, cattle, sheep, goat, fish, and pork are the most common meat sources globally. However, in a few nations, particularly in arid and semi-arid regions, camel meat is renowned as the primary source of animal protein that equals, and in some cases exceeds, the commercial importance of other meats (31, 71–74). Bones include up to 95% elastic protein, collagen fibers, and inorganic minerals such as calcium and phosphate, which help to prevent bone fracture. Ethnozoologists discovered that various species of animals, including the Indian gagata, horse, goat, fruit bat, deer, crow, crab-eating macaque, common carp, cinereous vulture, and alpine musk deer, were used to treat a variety of ailments, including wound healing, digestion, heart strength, ear ache, lumbago, skin, chest pain, and urine problems (18, 29, 55, 75–84).

Nanoparticles can operate as carriers for fish oil ingredients (85), preserving them from degradation in the gastrointestinal system and allowing for regulated release at particular locations throughout the body. Several forms of nanoparticles, including liposomes (86), nanoemulsions (87), and polymeric nanoparticles (88), have been investigated for encapsulating fish oil or its active components, such as eicosapentaenoic acid (89) and docosahexaenoic acid (90). Docosahexaenoic acid has received a lot of interest in the field of nanomedicine because of its potential health advantages and therapeutic qualities. These fatty acids play important roles in a variety of physiological processes, including cancer (91), inflammation (92), and diabetic problems (93). Omega-3 fatty acids in vertebrate fats have been shown to reduce inflammation. Ethnobiologists discovered that lipids are utilized to treat neurological disorders, atherosclerosis, thrombosis, and the effects of aging (32, 94–96). The previous published data showed that fats of various animals species, i.e., wild boar (*Sus scrofa*), turtle (Aspideretes sp.), streaked prochilod (*Prochilodus platensis*), sheep (*Ovis* sp.), mongoose (*Herpestes* sp.), bat (*Pteropus* sp.), lizard (*Hemidactylus* sp.), Irrawaddy, dolphin (*Orcaella brevirostris*), Indus Valley spiny-tail ground lizard (*Saara hardwickii*), Indian rock python (*Python molurus*), Indian flap-shelled turtle (*Lissemys punctata andersoni*), Indian bullfrog (*Hoplobatrachus tigerinus*), horse (*Equus* sp.), Himalayan Serow (*Capricornis thar*), hen (*Gallus* sp.), jackal (*Canis* sp.), hare (*Lepus* sp.), green pond frog (*Euphlyctis Hexadactylus*), goat (*Capra aegagrus hircus*), deer (Cervidae), cow (Bos sp.), common leopard gecko (*Eublepharis macularius*), cat (*Felis* sp.), buffalo (*Bubalus bubalis*) and Asiatic black bear (*Ursus* sp.), are used to cure different ailments such as wounds and injuries, toothache (97), wound skin burn and crack, pain in back (26, 98), sexual stimulant (18, 26, 29, 82, 97, 99, 100), rheumatism (26, 82), pain in muscles (82, 83), menstruation problem (84), asthma (26, 82, 83), impotency (18, 29), head pain (98), erysipelas (83), ear disease, wounds (84, 97), cancer (29), asthma, wart (26, 97), arthritis (26), anemia, fever, paralysis (26, 83), allergy, wound, skin disease (26, 76, 83), allergy, typhoid, ear infection (82, 101), and pain in joints (100).

Milk is one of the most important and oldest foods. Mammalian species’ milk consists of lactose, ash, fats, proteins, solids, and water (102–111). Milk of different mammalian species, i.e., *Panthera tigris*, *Ovis aries*, *Muntiacus muntjak*, *Homo sapiens*, *Equus caballus*, *Equus asinus*, *Equus africanus*, *Capra hircus*, *Capra aegagrus*, *Camelus dromedaries*, *Bubalus bubalis*, and *Bos taurus*, is used to cure different sicknesses, i.e., enhance immunity, wound, whooping cough, skin burn, sexual power, red eyes, pain, muscular pain, jaundice, invigorative, hiccup, hepatitis, headache, gastritis, eye, diabetes, cough, cold, catarrh, and arthritis (7, 18, 26, 82, 84, 112–126).

The egg is a good source of protein and nutrients for people and a source of chemicals and elements such as “phosphorus,” “selenium,” “amino acid,” “iron, vitamins A, B6, B12,” and “folic acid.” Asthma, high blood pressure, breast cancer, bronchitis, burns, CNS, cold, diabetes, eye diseases, fever, hemorrhoids, indigestion, jaundice, mental disorders, night blindness, nourishment, sinusitis, sprains, tooth, weak eye side, weakness, and weight loss are all treated with eggs (18, 26, 37, 114, 115, 119–121, 124, 127–138). The egg has components that provide the best environment for an embryo’s development and growth. Except for vitamin C, it is a major source of important nutrients for humans. Eggs are an incredibly tasty and healthy item that can be utilized in a variety of ways (37).

Feather is utilized as a biomaterial since it is inexpensive and environmentally beneficial. Feathers are made up of “α-helix” and “β-sheet.” Bird feathers are utilized for decoration and as toys. Feathers of various species are used in traditional medicine, e.g., *Phalacrocorax brasilianus*, *Nothura boraquira*, *Meleagris gallopayo*, *Coryus splendens*, *Corythaeola cristata*, *Coragyps atratus*, *Columba livia*, and *Ceryle rudis*, to treat alcoholism, asthma, cough, cough, flu, headache, and typhoid (49, 82, 112, 113, 119, 121, 124, 133, 139–142). Feathers are used for a variety of purposes, such as antibacterial activity modification, biosorbents, cell viability enhancement, cosmetic micro- and nanoparticles, and wound dressing in industry. Graphene Oxide is utilized as a bio-composites, bio-fertilizer, biomaterial, feeding supplement, bioplastic, electrode material, fire-resistant substance, leather processing, paper formation, protein for ruminants, regenerated fibers, textile fibers, thermoplastic films, tissue reformation, and wound healing (49, 143–164).

Honey is composed of amino acids (165, 166), minerals (167), organic acids, phenolic compounds (168), solid particles (169), sugars (170), vitamins (171), water, proteins, disaccharides (172–174), and volatile compounds (175). Honey is utilized as a therapy in folk medicine to heal acidity, obesity, allergy, Alzheimer’s disease, asthma, atherosclerosis, burn, cancer, cold, cough, diabetes mellitus, diarrhea, expectorant, eye infection, gastritis, hypertension, influenza, migraine, skin, snake-bite, spleen, throat pain, tonsils, toothache, and urinary system (7, 18, 30, 81, 82, 84, 98, 115–117, 119, 120, 123, 124, 126, 137, 176–183). Honey is also used in nanomedicine to heal diverse sicknesses and behaves as oxidative stress, heart, blood pressure, anti-proliferative, antioxidant, anti-inflammatory, anti-fungal endophthalmitis, anti-diabetic, anti-cataract, antibiotic, antibacterial, and anti-apoptosis (184–199). Many fauna species have been shown to be quite adaptable in their applications. The maximum relative significance levels may indicate that animals are easily accessible and affordable (200–202).

## 5 Conclusion

The folklore animal-based medicinal concept of Gujranwala communities indicates that people have a strong link with ecology. The ethnopharmacological benefits of the fauna in Gujranwala were documented for the first time. In addition, 54 fauna species are employed to treat various human ailments. Different body parts of animals (*viz.* skin, fat, brain, meat, milk, blood, hair, saliva, flesh, liver, scale, tail, egg, feather, honey, and whole body) are used to treat various diseases such as skin infection, sexual problems, pain in the backbone, joint pain, eyesight, night blindness, enhance immunity and memory, cold, weakness, body pain, burn, smallpox, wounds in feet, engulf of poisonous things, pain in muscles, arthritis, diabetes, fever, epilepsy, allergy, asthma, burn, herpes, ear pain, paralysis, cough, swelling, cancer, bronchitis, girls maturity, stomach, and antibacterial infections. The present results provide data that may be constructive for the conservation of fauna in the district of Gujranwala, Punjab, Pakistan. Screening of bioactive materials and “*in vivo”* and/or “*in vitro”* studies of the zoological activities of the species with the highest FC, FL, RPL, and ROP, may be significant for wild animal-based novel medications. Due to financial constraints, the local population lives in isolated, hilly villages that are far from urban areas, primarily involved in agricultural labor, home-based businesses, and livestock rearing. The locals heavily rely on medicinal flora to meet their basic medical needs. While nearby regions such as Malakand, Dir Lower, Chitral, and Swat have been thoroughly investigated for their medicinal plants, this study focuses specifically on Dir Upper. This investigation supported the theory that the local indigenous knowledge would differ significantly from its surroundings. Notably, 80% of people in developing nations rely on herbal health remedies. The primary healthcare needs are met in large part by local healers. Medicinal plants with significant UV protection were found to protect the studied area’s biodiversity. Unfortunately, anthropogenic practices, such as overharvesting, overpopulation, and grazing, endanger regional biodiversity. To stop the impending extinction of medicinal plants in the study area, initiatives for cultivating these species must be implemented immediately. This strategy will lessen the risks brought on by human activity and maintain the availability of vital plant resources for future generations.

## Data availability statement

The original contributions presented in the study are included in the article/supplementary material, further inquiries can be directed to the corresponding authors.

## Author contributions

AK: Conceptualization, Data curation, Formal analysis, Methodology, Writing – original draft. MA: Conceptualization, Investigation, Methodology, Project administration, Writing – original draft. TH: Project administration, Supervision, Validation, Writing – review & editing. AA: Resources, Validation, Writing – review & editing. Z-nM: Resources, Validation, Writing – review & editing. AH: Data curation, Validation, Writing – review & editing. SA: Data curation, Writing – original draft. US: Data curation, Investigation, Writing – review & editing. MSA: Supervision, Validation, Visualization, Writing – review & editing. MM: Funding acquisition, Project administration, Resources, Validation, Visualization, Writing – review & editing. MH: Validation, Methodology, Writing – review & editing. RB: Supervision, Validation, Visualization, Writing – review & editing. AMA: Conceptualization, Project administration, Supervision, Validation, Visualization, Writing – review & editing. MA-Y: Validation, Visualization, Writing – review & editing. HE: Validation, Visualization, Writing – review & editing. EM: Validation, Visualization, Writing – review & editing. MHH: Methodology, Data curation, Writing – original draft preparation, Writing – review and editing.

## References

[1] Faiz M Adil S Nawaz A. Chemical composition, ethnopharmacological applications of animal biles-mini review. J Wildlife Ecol. (2022) 6:172–7.

[2] Altaf M. Assessment of avian and mammalian diversity at selected sites along river Chenab. Lahore, Pakistan: University of Veterinary and Animal Sciences (2016).

[3] Costa-Neto EM. Animal-based medicines: biological prospection and the sustainable use of zootherapeutic resources. An Acad Bras Cienc. (2005) 77:33–43. doi: 10.1590/S0001-37652005000100004, PMID: 15692677

[4] Holennavar P. Use of animal and animal derived products as medicines by the inhabitants of villages in Athani taluka of Belagavi District. Karnataka Int J App Res. (2015) 1:437–40. doi: 10.1007/s10668-019-00404-6

[5] Kassam A. World resources 2000–2001: people and ecosystems: the fraying web of life. Washington, DC: World resources institute (2000) (2002). 389 p.

[6] Lawal O Banjo A. Survey for the usage of arthropods in traditional medicine in southwestern Nigeria. J Entomol. (2007) 4:104–12. doi: 10.3923/je.2007.104.112

[7] Lev E. Traditional healing with animals (zootherapy): medieval to present-day Levantine practice. J Ethnopharmacol. (2003) 85:107–18. doi: 10.1016/S0378-8741(02)00377-X 12576209

[8] Singh V. A note on the use of wild animal organs in Tibetan medicine. Traffic India. Lodhi Estate, New Delhi: WWF (2000).

[9] Still J. Use of animal products in traditional Chinese medicine: environmental impact and health hazards. Complement Ther Med. (2003) 11:118–22. doi: 10.1016/S0965-2299(03)00055-4, PMID: 12801499

[10] Unnikrishnan PM. Animals in ayurveda. Amruth. (1998):1–15.

[11] Arif U Bhatti KH Ajaib M Wagay NA Majeed M Zeb J. Ethnobotanical indigenous knowledge of tehsil Charhoi, district Kotli, Azad Jammu and Kashmir, Pakistan. Ethnobot Res Appl. (2021) 22:1–24. doi: 10.32859/era.22.50.1-24

[12] Arshad F Waheed M Harun N Fatima K Khan BA Fatima K. Indigenous farmer's perception about fodder and foraging species of semi-arid lowlands of Pakistan: A case study of district Kasur Pakistan. Taiwania. (2022) 67:510–23. doi: 10.6165/tai.2022.67.510

[13] Bashir SM Altaf M Hussain T Umair M Majeed M Mangrio WM. Vernacular taxonomy, cultural and ethnopharmacological applications of avian and mammalian species in the vicinity of Ayubia National Park. Himalayan Region Biol. (2023) 12:609. doi: 10.3390/biology12040609 PMC10135773 37106809

[14] Haq SM Yaqoob U Majeed M Amjad MS Hassan M Ahmad R. Quantitative ethnoveterinary study on plant resource utilization by indigenous communities in high-altitude regions. Front Vet Sci. (2022) 9:944046. doi: 10.3389/fvets.2022.944046, PMID: 36277063 PMC9583879

[15] Majeed M Lu L Anwar MM Tariq A Qin S El-Hefnawy ME. Prediction of flash flood susceptibility using integrating analytic hierarchy process (AHP) and frequency ratio (FR) algorithms. Front Environ Sci. (2023) 10:1037547. doi: 10.3389/fenvs.2022.1037547

[16] Majeed M Tariq A Haq SM Waheed M Anwar MM Li Q. A detailed ecological exploration of the distribution patterns of wild Poaceae from the Jhelum District (Punjab). Pakistan Sustainability. (2022) 14:3786. doi: 10.3390/su14073786

[17] Saeed A Umair M Altaf M Hussain T Abbasi AM. Ethno-veterinary medicines of South Punjab. Pakistan J Wildlife Ecol. (2022) 6:64–81.

[18] Altaf M Umair M Abbasi AR Muhammad N Abbasi AM. Ethnomedicinal applications of animal species by the local communities of Punjab Pakistan. J Ethnobiol Ethnomed. (2018) 14:55. doi: 10.1186/s13002-018-0253-4, PMID: 30111346 PMC6094468

[19] Alves RR Rosa IL. Zootherapy goes to town: the use of animal-based remedies in urban areas of NE and N Brazil. J Ethnopharmacol. (2007) 113:541–55. doi: 10.1016/j.jep.2007.07.015, PMID: 17719192

[20] Mirza ZB Wasiq H. A field guide to birds of Pakistan Bookland. Lahore: (2007).

[21] Roberts TJ. The mammals of Pakistan. New Yark: Oxford University Press (1997).

[22] Khan MS. Checklist of amphibians of Pakistan. Pakistan J Wildlife. (2010) 1

[23] Khan MS. Amphibian and reptiles of Pakistan. Pakistan: Krieger Publisher Company (2006).

[24] Altaf M. Study of avian diversity in urban areas of Gujranwala, Pakistan. Int J of. (2021)

[25] Altaf M. Assessment of avian and mammalian diversity at selected sites along river Chenab. Lahore-Pakistan: University of Veterinary and Animal Sciences (2016).

[26] Altaf M Javid A Umair M Iqbal KJ Rasheed Z Abbasi AM. Ethnomedicinal and cultural practices of mammals and birds in the vicinity of river Chenab Punjab-Pakistan. J Ethnobiol Ethnomed. (2017) 13:1–24. 28701165 10.1186/s13002-017-0168-5 PMC5508792

[27] Altaf M Khan AM Umair M Irfan MMA. Status of wild birds and mammals in urban habitats of Gujranwala, Punjab Pakistan. Punjab Univ J Zool. (2012) 27:9–12.

[28] Altaf M Javid A Khan AM Hussain A Umair M Ali Z. The status of fish diversity of river Chenab, Pakistan. J Animal & Plant Sci. (2015) 25:564–9.doi: 10.1186/s13002-017-0168-5

[29] Altaf M Abbasi AM Umair M Amjad MS Irshad K Khan AM. The use of fish and herptiles in traditional folk therapies in three districts of Chenab riverine area in Punjab. Pakistan J Ethnobiol Ethnomed. (2020) 16:1–21. doi: 10.1186/s13002-020-00379-z PMC7313147 32580733

[30] Altaf M Umair M. Diversity, distribution and medicinal importance of honeybees in the world-A review. J Wildlife Ecol. (2020) 4:130–41.

[31] Haidar R Bashir SM. Chemical composition, traditional and modern uses of meat of animals-a review. J Wildlife Ecol. (2021) 5:47–55.

[32] Ijaz S Faiz M. Chemical composition, folk and modern uses of fats and oil-a review. J Wildlife Ecol. (2021) 5:104–10.

[33] Khoja AA Haq SM Majeed M Hassan M Waheed M Yaqoob U. Diversity, ecological and traditional knowledge of pteridophytes in the western Himalayas. Diversity. (2022) 14:628. doi: 10.3390/d14080628

[34] Majeed M Bhatti KH Amjad MS. Impact of climatic variations on the flowering phenology of plant species in JHELUM district, Punjab Pakistan. Appl Ecol Environ Res. (2021) 19:3343–76. doi: 10.15666/aeer/1905_33433376

[35] Majeed M Bhatti KH Pieroni A Sõukand R Bussmann RW Khan AM. Gathered wild food plants among diverse religious groups in Jhelum District, Punjab, Pakistan. Food Secur. (2021) 10:594. doi: 10.3390/foods10030594 PMC7999103 33799901

[36] Majeed M Tariq A Anwar MM Khan AM Arshad F Mumtaz F. Monitoring of land use–land cover change and potential causal factors of climate change in Jhelum district, Punjab, Pakistan, through GIS and multi-temporal satellite data. Landscape. (2021) 10:1026. doi: 10.3390/land10101026

[37] Tariq S. Chemical composition and traditional uses of eggs of different avian species-A review. J Wildlife Ecol. (2020) 4:45–50.

[38] Akhtar S. Antimicrobial effect of silver nanoparticles synthesized from skin of *Laudakia agrorensis*. J Wildlife Ecol. (2021) 5:176–85.

[39] Altaf M Faiz M. Snake venom-a review. J Wildlife Ecol. (2021) 5:146–58.

[40] Habib S. Antibacterial activity of biogenic synthesized silver nanoparticles using skin of Kashmir Nadi frog *Paa barmoachensis*. J Wildlife Ecol. (2022) 6:07–12.

[41] Nazir N. Biosynthesis of silver nanoparticles from skin of Hazara toad (*Bufo melanostictus*) and assessment of antibacterial activity. J Wildlife Ecol. (2021) 5:111–9.

[42] Zainab S. Antibacterial and antibiofilm activity of bull frog *Hoplobatrachus tigerinus* skin extract. J Wildlife Ecol. (2021) 5:32–7.

[43] Khan MSH Ullah S Hamed MH Altaf M. A study of illegal wildlife trade and seizures in Pakistan. J Wildlife Ecol. (2020) 4:193–210.

[44] Alves RR. Relationships between fauna and people and the role of ethnozoology in animal conservation. Ethnobiol Conserv. (2012) 1:1–69. doi: 10.15451/ec2012-8-1.2-1-69

[45] Alves RRN Silva JS da Silva CL Albuquerque UP. Ethnozoology and animal conservation. In: Ethnozoology Elsevier. (2018):481–96. doi: 10.1016/B978-0-12-809913-1.00025-9

[46] Boivin NL Zeder MA Fuller DQ Crowther A Larson G Erlandson JM. Ecological consequences of human niche construction: examining long-term anthropogenic shaping of global species distributions. Proc Natl Acad Sci. (2016) 113:6388–96. doi: 10.1073/pnas.1525200113, PMID: 27274046 PMC4988612

[47] Dickman AJ. Complexities of conflict: the importance of considering social factors for effectively resolving human–wildlife conflict. Animal. (2010) 13:458–66. doi: 10.1111/j.1469-1795.2010.00368.x

[48] Saunders CD. The emerging field of conservation psychology. Hum Ecol Rev. (2003):137–49.

[49] Adil S Tariq S. Study of traditional and modern applications of feathers-a review. J Wildlife Ecol. (2020) 4:141–50.

[50] Ali A Khan MSH Altaf M. Winter survey of birds at district of the Badin, Pakistan. J Wildlife Ecol. (2018) 2:11–22.

[51] Altaf M Abbasi AM Umair M Amjad MS Muhammad N Iqbal KJ. The usage of freshwater fishes in cultural and folklore therapies among the people along river Jhelum, Punjab Pakistan. J Wildlife Ecol. (2021) 5:79–99.

[52] Aslam H Faiz M. Ethnopharmacological and modern applications of milk of various mammalian species-a review. J Wildlife Ecol. (2020) 4:211–26.

[53] Hassan M Haq SM Ahmad R Majeed M Sahito HA Shirani M. Traditional use of wild and domestic Fauna among different ethnic groups in the Western Himalayas—A cross cultural analysis. Animals. (2022) 12:2276. doi: 10.3390/ani12172276, PMID: 36077997 PMC9454963

[54] Hassan M Haq SM Majeed M Umair M Sahito HA Shirani M. Traditional food and medicine: Ethno-traditional usage of fish Fauna across the valley of Kashmir: A western Himalayan region. Diversity. (2022) 14:455. doi: 10.3390/d14060455

[55] Ijaz S Iftikhar A. Chemical composition, ethnomedicinal and industrial uses of bones-a review. J Wildlife Ecol. (2021) 5:56–9.

[56] Khan AM Li Q Saqib Z Khan N Habib T Khalid N. MaxEnt modelling and impact of climate change on habitat suitability variations of economically important chilgoza pine (*Pinus gerardiana* wall.) in South Asia. Forests. (2022) 13:715. doi: 10.3390/f13050715

[57] Majeed M Khan AM Habib T Anwar MM Sahito HA Khan N. Vegetation analysis and environmental indicators of an arid tropical forest ecosystem of Pakistan. Ecol Indic. (2022) 142:109291. doi: 10.1016/j.ecolind.2022.109291

[58] Majeed M Lu L Haq SM Waheed M Sahito HA Fatima S. Spatiotemporal distribution patterns of climbers along an abiotic gradient in Jhelum district, Punjab. Pakistan Forests. (2022) 13:1244. doi: 10.3390/f13081244

[59] Mughal S Pervaz M Bashir SM Shamashad SS. Assessment of diversity and ethnopharmacological uses of birds in chakar, Hattian Bala district, Azad Jammu and Kashmir-Pakistan. J Wildlife Ecol. (2020) 4:35–44.

[60] Noor U Haider R. Assessment of herpetofauna diversity and human-herpetofauna-interaction in district Sudhnoti, Azad Jammu and Kashmir Pakistan. J Wildlife Ecol. (2020) 4:156–63.

[61] Saleem R Altaf M Umair M Amjad MS Abbasi AM. Ethnopharmacological applications of the amphibians and reptiles among the people in the vicinity of Margalla Hill National Park, Islamabad Pakistan. J Wildlife Ecol. (2021) 5:13–25.

[62] Chattha SA Malik MF Altaf M Mahmood S Khan J Ali A. Human pursuits cause of road killing of wild and domestic animals by accident on National Highway of Punjab Pakistan. J Wildlife Ecol. (2017) 1:8–16.

[63] Hakeem F Altaf M Manzoor S Rauf K Mumtaz B Bashir M. Assessment of behavioral study, human activities impacts and interaction with streak laughingthrush (*Trochalopteron lineatum*) in district Bagh, Azad Jammu and Kashmir-Pakistan. J Wildlife Ecol. (2017) 1:1–7.

[64] Muhammad N Khan AM Umair M Qazi A Yaqoob M Ashraf S. Assessment of distribution and ethnocultural uses of the sol (*Channa marulius*) in Punjab. Pakistan J Wildlife Ecol. (2017) 1:35–41.

[65] Alexiades MN Sheldon JW. Selected guidelines for ethnobotanical research: A field manual. New York: Botanical Garden (1996).

[66] Friedman J Yaniv Z Dafni A Palewitch D. A preliminary classification of the healing potential of medicinal plants, based on a rational analysis of an ethnopharmacological field survey among Bedouins in the Negev Desert, Israel. J Ethnopharmacol. (1986) 16:275–87. doi: 10.1016/0378-8741(86)90094-2, PMID: 3747566

[67] Ali-Shtayeh MS Yaniv Z Mahajna J. Ethnobotanical survey in the Palestinian area: a classification of the healing potential of medicinal plants. J Ethnopharmacol. (2000) 73:221–32. doi: 10.1016/S0378-8741(00)00316-0, PMID: 11025160

[68] Hui YH. Handbook of meat and meat processing. US: CRC press (2012).

[69] Keeton JT Eddy S. Chemical composition In: Yclopedia of meat sciences: Enc. Oxford: Elsevier Academic Press (2004)

[70] Cheung PCK Mehta BM. Handbook of food chemistry. Berlin Heidelberg: Springer (2015).

[71] Abrhaley A Leta S. Medicinal value of camel milk and meat. J Appl Anim Res. (2018) 46:552–8. doi: 10.1080/09712119.2017.1357562

[72] Faiz M. Folklore or facts: investigating the nutritional and ethnopharmacological roles of animal parts. J Wildlife Ecol. (2023) 7:211–6.

[73] Schönfeldt HC Gibson N. Changes in the nutrient quality of meat in an obesity context. Meat Sci. (2008) 80:20–7. doi: 10.1016/j.meatsci.2008.05.025, PMID: 22063166

[74] Williams P. Nutritional composition of red meat. Nutr Diet. (2007) 64:S113–9. doi: 10.1111/j.1747-0080.2007.00197.x

[75] Altaf M Altaf M Khichi TAA. Cultural significance and medicinal applications of herpetofauna in Bagh, Azad Jammu and Kashmir, Pakistan. J WIldlife Ecol. (2023) 7

[76] Bullitta S Re GA Manunta MDI Piluzza G. Traditional knowledge about plant, animal, and mineral-based remedies to treat cattle, pigs, horses, and other domestic animals in the Mediterranean island of Sardinia. J Ethnobiol Ethnomed. (2018) 14:50. doi: 10.1186/s13002-018-0250-7, PMID: 30029686 PMC6054737

[77] Ghosh T Singhamahapatra R Mandal F. Traditional use of animals among Santhals of Bankura district. Int J Latest Res Sci Technol. (2013) 2:95–6.

[78] Hall J. Textbook of medical physiology. Philadelphia: Elsevier (2011).

[79] Hussain T Altaf M. Ethnobiology of Cholistan. Pakistan: PEACE International Publisher (2023).

[80] Muhammad N Altaf M Abbasi AM Khan AM Iqbal KJ. From water to remedy: fishes as ethnopharmacological resources along the river Ravi, Punjab, Pakistan. J WIldlife Ecol. (2023) 7

[81] Vallejo JR González JA. Fish-based remedies in Spanish ethnomedicine: a review from a historical perspective. J Ethnobiol Ethnomed. (2014) 10. doi: 10.1186/1746-4269-10-37 PMC4025190 24885245

[82] Vijayakumar S Yabesh JM Prabhu S Ayyanar M Damodaran R. Ethnozoological study of animals used by traditional healers in Silent Valley of Kerala, India. J Ethnopharmacol. (2015) 162:296–305. doi: 10.1016/j.jep.2014.12.055 25571847

[83] Vijayakumar S Prabhu S Yabesh JM Prakashraj R. A quantitative ethnozoological study of traditionally used animals in Pachamalai hills of Tamil Nadu, India. J Ethnopharmacol. (2015) 171:51–63. doi: 10.1016/j.jep.2015.05.023 26002766

[84] Yeshi K Morisco P Wangchuk P. Animal-derived natural products of Sowa Rigpa medicine: their pharmacopoeial description, current utilization and zoological identification. J Ethnopharmacol. (2017) 207:192–202. doi: 10.1016/j.jep.2017.06.009 28606809

[85] Kadakia E Harpude P Parayath N Bottino D Amiji M. Challenging the CNS targeting potential of systemically administered nanoemulsion delivery systems: a case study with rapamycin-containing fish oil nanoemulsions in mice. Pharm Res. (2019) 36:1–12. doi: 10.1007/s11095-019-2667-7 31297653

[86] Hadian Z. A review of nanoliposomal delivery system for stabilization of bioactive omega-3 fatty acids. Electron Physician. (2016) 8:1776–85. doi: 10.19082/1776, PMID: 26955449 PMC4768928

[87] Khoshbou Lahijani L Ahari H Sharifan A. Enhancement of food safety using nanoemulsion with emphasize on fish food: A review. Sustain Aquacul Health Manag J. (2019) 5:26–44.

[88] Miyazawa T Itaya M Burdeos GC Nakagawa K Miyazawa T. A critical review of the use of surfactant-coated nanoparticles in nanomedicine and food nanotechnology. Int J Nanomedicine. (2021) 16:3937–99. doi: 10.2147/IJN.S298606 34140768 PMC8203100

[89] Escobar-García JD Prieto C Pardo-Figuerez M Lagaron JM. Room temperature nanoencapsulation of bioactive eicosapentaenoic acid rich oil within whey protein microparticles. Nano. (2021) 11:575. doi: 10.3390/nano11030575, PMID: 33668857 PMC7996356

[90] Guo P Si M Wu D Xue HY Hu W Wong HL. Incorporation of docosahexaenoic acid (DHA) enhances nanodelivery of antiretroviral across the blood-brain barrier for treatment of HIV reservoir in brain. J Control Release. (2020) 328:696–709. doi: 10.1016/j.jconrel.2020.09.050, PMID: 33010335 PMC7749038

[91] Wen X Reynolds L Mulik RS Kim SY Van Treuren T Nguyen LH. Hepatic arterial infusion of low-density lipoprotein docosahexaenoic acid nanoparticles selectively disrupts redox balance in hepatoma cells and reduces growth of orthotopic liver tumors in rats. Gastroenterology. (2016) 150:488–98. doi: 10.1053/j.gastro.2015.10.008, PMID: 26484708 PMC4727982

[92] Alaarg A Jordan NY Verhoef JJ Metselaar JM Storm G Kok RJ. Docosahexaenoic acid liposomes for targeting chronic inflammatory diseases and cancer: an in vitro assessment. Int J Nanomedicine. (2016) 11:5027–40. doi: 10.2147/IJN.S115995, PMID: 27785012 PMC5063558

[93] Hussein J Rasheed W Ramzy T Nabeeh M Harvy M El-Toukhy S. Synthesis of docosahexaenoic acid–loaded silver nanoparticles for improving endothelial dysfunctions in experimental diabetes. Hum Exp Toxicol. (2019) 38:962–73. doi: 10.1177/0960327119843586, PMID: 31018711

[94] Breteler MM. Vascular risk factors for Alzheimer’s disease: an epidemiologic perspective. Neurobiol Aging. (2000) 21:153–60. doi: 10.1016/S0197-4580(99)00110-4 10867200

[95] Haag M. Essential fatty acids and the brain. Can J Psychiatr. (2003) 48:195–203. doi: 10.1177/070674370304800308 12728744

[96] Wilson L. Fats and oils for optimum health The Center for Development (2015).

[97] Kendie FA Mekuriaw SA Dagnew MA. Ethnozoological study of traditional medicinal appreciation of animals and their products among the indigenous people of Metema woreda, North-Western Ethiopia. J Ethnobiol Ethnomed. (2018) 14:37. doi: 10.1186/s13002-018-0234-7, PMID: 29792196 PMC5967044

[98] Sreekeesoon DP Mahomoodally MF. Ethnopharmacological analysis of medicinal plants and animals used in the treatment and management of pain in Mauritius. J Ethnopharmacol. (2014) 157:181–200. doi: 10.1016/j.jep.2014.09.030 25261690

[99] Haq SM Calixto ES Yaqoob U Ahmed R Mahmoud AH Bussmann RW. Traditional usage of wild Fauna among the local inhabitants of Ladakh. Trans-Himalayan Region Animals. (2020) 10:2317. 33297401 10.3390/ani10122317 PMC7762308

[100] Nijman V Shepherd CR. Ethnozoological assessment of animals used by Mon traditional medicine vendors at Kyaiktiyo, Myanmar. J Ethnopharmacol. (2017) 206:101–6. doi: 10.1016/j.jep.2017.05.010 28506903

[101] Kim H Song M-J. Analysis of ethnomedicinal practices for treating skin diseases in communities on Jeju Island (Korea). Indian J Tradit Knowl. (2014) 13:673–80.

[102] Abdullahi A. Camel Milk-A Review. J Animal Sci Livestock Production. (2019) 3:13–8.

[103] Ballard O Morrow AL. Human milk composition: nutrients and bioactive factors. Pediatr Clin. (2013) 60:49–74. 10.1016/j.pcl.2012.10.002 PMC3586783 23178060

[104] Getaneh G Mebrat A Wubie A Kendie H. Review on goat milk composition and its nutritive value. J Nutrs Health Sci. (2016) 3:401–9.

[105] Grădinaru AC Creangă Ş Solcan G. Milk–a review on its synthesis, composition, and quality assurance in dairy industry. Human Vet Med. (2015) 7:173–7.

[106] Guo H Pang K Zhang X Zhao L Chen S Dong M. Composition, physiochemical properties, nitrogen fraction distribution, and amino acid profile of donkey milk. Joint annual meeting abstracts. (2007) 90:1635–43. doi: 10.3168/jds.2006-600, PMID: 17369203

[107] Hamad M Baiomy A. Physical properties and chemical composition of cow's and buffalo's milk in Qena governorate. J food and Dairy Sci Mansoura Uni. (2010) 1:397–403. doi: 10.21608/jfds.2010.82466

[108] Kula JT Tegegne D. Chemical composition and medicinal values of camel milk. Int J Res Stud Biosci. (2016) 4:13–25.

[109] Merlin Junior IA Sifuentes dos Santos J Grecco Costa L Grecco Costa R Ludovico A de Almeida Rego FC. Sheep milk: physical-chemical characteristics and microbiological quality. Arch Latinoam Nutr. (2015) 65:193–8. 26821492

[110] Spreer E. Milk and dairy product technology. US: CRC Press (1998).

[111] Wiley AS. Re-imagining milk: Cultural and biological perspectives. UK: Routledge (2015).

[112] Alonso-Castro AJ. Use of medicinal fauna in Mexican traditional medicine. J Ethnopharmacol. (2014) 152:53–70. doi: 10.1016/j.jep.2014.01.005, PMID: 24440438

[113] Alves RR Neto NAL Brooks SE Albuquerque UP. Commercialization of animal-derived remedies as complementary medicine in the semi-arid region of Northeastern Brazil. J Ethnopharmacol. (2009) 124:600–8. doi: 10.1016/j.jep.2009.04.049, PMID: 19422902

[114] Alves RRN Neta ROS Trovão D Barbosa J Barros AT Dias TLP. Traditional uses of medicinal animals in the semi-arid region of northeastern Brazil. J Ethnobiol Ethnomed. (2012) 8:4269–8. doi: 10.1186/1746-4269-8-41 PMC3547754 23050756

[115] Barros FB Varela SA Pereira HM Vicente L. Medicinal use of fauna by a traditional community in the Brazilian Amazonia. J Ethnobiol Ethnomed. (2012) 8:37. doi: 10.1186/1746-4269-8-37, PMID: 23013927 PMC3502351

[116] Benítez G. Animals used for medicinal and magico-religious purposes in western Granada Province, Andalusia (Spain). J Ethnopharmacol. (2011) 137:1113–23. doi: 10.1016/j.jep.2011.07.036, PMID: 21801827

[117] Betlloch Mas I Chiner E Chiner Betlloch J Llorca FX Martín Pascual L. (2014). The use of animals in medicine of Latin tradition: Study of the Tresor de Beutat, a medieval treatise devoted to female cosmetics: Photon.

[118] Borah MP Prasad SB. Ethnozoological study of animals based medicine used by traditional healers and indigenous inhabitants in the adjoining areas of gibbon wildlife sanctuary, Assam. India J ethnobiol ethnome. (2017) 13:39. doi: 10.1186/s13002-017-0167-6, PMID: 28666483 PMC5493085

[119] Haileselasie TH. Traditional zootherapeutic studies in Degu’a Tembien, northern Ethiopia. Curr Res J Biol Sci. (2012) 4:563–9.

[120] Lohani U. Traditional uses of animals among jirels of Central Nepal. Ethno Med. (2011) 5:115–24. doi: 10.1080/09735070.2011.11886398

[121] Martínez GJ. Use of fauna in the traditional medicine of native Toba (Qom) from the Argentine Gran Chaco region: an ethnozoological and conservationist approach. Ethnobiol Conserv. (2013) 2:1–43. doi: 10.15451/ec2013-8-2.2-1-43

[122] Mishra N Rout S Panda T. Ethno-zoological studies and medicinal values of Similipal biosphere reserve, Orissa, India. Afr J Pharm Pharmacol. (2011) 5:6–11.

[123] Mootoosamy A Mahomoodally MF. A quantitative ethnozoological assessment of traditionally used animal-based therapies in the tropical island of Mauritius. J Ethnopharmacol. (2014) 154:847–57. doi: 10.1016/j.jep.2014.05.001 24824877

[124] Padmanabhan P Sujana K. Animal products in traditional medicine from Attappady hills of Western Ghats. Indian J Tradit Knowl. (2008) 7:326–9.

[125] WHO. Technical updates of the guidelines on integrated Management of Childhood Illness (IMCI). Geneva: Evidence and recommendations for further adaptations (2005).

[126] Yirga G Teferi M Gebreslassea Y. Ethnozoological study of traditional medicinal animals used by the people of Kafta-Humera District, northern Ethiopia. Int J Med Medical Sci. (2011) 3:316–20.

[127] Alonso-Castro AJ Carranza-Álvarez C Maldonado-Miranda JJ del Rosario Jacobo-Salcedo M Quezada-Rivera DA Lorenzo-Márquez H. Zootherapeutic practices in Aquismón, San Luis Potosí, México. J Ethnopharmacol. (2011) 138:233–7. doi: 10.1016/j.jep.2011.09.020, PMID: 21963568

[128] Alves RRN Oliveira MGG Barboza RRD Lopez LCS Oliveira MGG. An ethnozoological survey of medicinal animals commercialized in the markets of Campina GrandeNE Brazil. Hum Ecol Rev. (2010) 17:11–7.

[129] Bagde N Jain S. An ethnozoological studies and medicinal values of vertebrate origin in the adjoining areas of Pench National Park of Chhindwara District of Madhya Pradesh, India. Int J lifesci. (2013) 1:278–83.

[130] Bagde N Jain S. Study of traditional man-animal relationship in CHHINDWARA district of Madhya Pradesh India. J Global Biosci. (2015) 4:1456–63.

[131] Betlu ALS. Indigenous knowledge of zootherapeutic use among the Biate tribe of Dima Hasao District, Assam, Northeastern India. J Ethnobiol Ethnomed. (2013) 9:1–16. 23938109 10.1186/1746-4269-9-56 PMC3751812

[132] Chellappandian M Pandikumar P Mutheeswaran S Paulraj MG Prabakaran S Duraipandiyan V. Documentation and quantitative analysis of local ethnozoological knowledge among traditional healers of Theni district, Tamil Nadu, India. J Ethnopharmacol. (2014) 154:116–30. doi: 10.1016/j.jep.2014.03.028, PMID: 24680989

[133] Jacobo-Salcedo M d R Alonso-Castro AJ Zarate-Martinez A. Folk medicinal use of fauna in Mapimi, Durango, México. J Ethnopharmacol. (2011) 133:902–6. doi: 10.1016/j.jep.2010.10.005, PMID: 20937375

[134] Dey A Gorai P Mukherjee A Dhan R Modak BK. Ethnobiological treatments of neurological conditions in the Chota Nagpur plateau, India. J Ethnopharmacol. (2017) 198:33–44. doi: 10.1016/j.jep.2016.12.040, PMID: 28017696

[135] Kim H Song M-J. Ethnozoological study of medicinal animals on Jeju Island, Korea. J Ethnopharmacol. (2013) 146:75–82. doi: 10.1016/j.jep.2012.11.011 23266277

[136] Lohani U. Man-animal relationships in Central Nepal. J Ethnobiol Ethnomed. (2010) 6. doi: 10.1186/1746-4269-6-31 PMC2987906 21050449

[137] Oliveira ES Torres DF Brooks SE Alves RR. The medicinal animal markets in the metropolitan region of Natal City, Northeastern Brazil. J Ethnopharmacol. (2010) 130:54–60. doi: 10.1016/j.jep.2010.04.010 20460145

[138] Souto WMS Barboza RRD da Silva MJ Alves RRN. Traditional knowledge of sertanejos about zootherapeutic practices used in ethnoveterinary medicine of NE Brazil. Indian J Tradit Knowl. (2012) 11:259–65.

[139] Bezerra DMM de Araujo HFP Alves A Alves RRN. Birds and people in semiarid northeastern Brazil: symbolic and medicinal relationships. J Ethnobiol Ethnomed. (2013) 9. doi: 10.1186/1746-4269-9-3, PMID: 23295130 PMC3599870

[140] Bobo KS Aghomo FFM Ntumwel BC. Wildlife use and the role of taboos in the conservation of wildlife around the Nkwende Hills Forest reserve; south-West Cameroon. J Ethnobiol Ethnomed. (2015) 11:2. doi: 10.1186/1746-4269-11-2, PMID: 25567094 PMC4326412

[141] dos Santos Soares VM de Lucena Soares HK da Silva SS de Lucena RFP. Local knowledge, use, and conservation of wild birds in the semi-arid region of Paraíba state, northeastern Brazil. J Ethnobiol Ethnomed. (2018) 14:77. doi: 10.1186/s13002-018-0276-x, PMID: 30514340 PMC6280514

[142] Lohani U. Eroding ethnozoological knowledge among Magars in Central Nepal. Indian J Tradit Knowl. (2011) 10:466–73.

[143] Amieva EJ-C Fuentes-Ramirez R Martinez-Hernandez A Millan-Chiu B Lopez-Marin LM Castaño V. Graphene oxide and reduced graphene oxide modification with polypeptide chains from chicken feather keratin. J Alloys Compd. (2015) 643:S137–43. doi: 10.1016/j.jallcom.2014.12.062

[144] Coward-Kelly G Chang VS Agbogbo FK Holtzapple MT. Lime treatment of keratinous materials for the generation of highly digestible animal feed: 1. Chicken feathers. Bioresources. (2006) 97:1337–43. doi: 10.1016/j.biortech.2005.05.021 16098740

[145] Flores-Hernández CG Colín-Cruz A Velasco-Santos C Castaño VM Rivera-Armenta JL Almendarez-Camarillo A. All green composites from fully renewable biopolymers: chitosan-starch reinforced with keratin from feathers. Polymers. (2014) 6:686–705. doi: 10.3390/polym6030686

[146] Gurav RG Jadhav JP. A novel source of biofertilizer from feather biomass for banana cultivation. Environ Sci Pollut Res. (2013) 20:4532–9. doi: 10.1007/s11356-012-1405-z, PMID: 23263761

[147] Karthikeyan R Balaji S Sehgal P. (2007). Industrial applications of keratins–A review.

[148] Khajavi R Rahimi MK Abbasipour M Brendjchi AH. Antibacterial nanofibrous scaffolds with lowered cytotoxicity using keratin extracted from quail feathers. J Bioact Compat Polym. (2016) 31:60–71. doi: 10.1177/0883911515598793

[149] Kumar SL Anandhavelu S Sivaraman J Swathy M. Modified extraction and characterization of keratin from Indian goat hoof: A biocompatible biomaterial for tissue regenerative applications. Integr Ferroelectr. (2017) 184:41–9. doi: 10.1080/10584587.2017.1368642

[150] Manivasagan P Sivakumar K Gnanam S Venkatesan J Kim S-K. Production, biochemical characterization and detergents application of keratinase from the marine actinobacterium actinoalloteichus sp. MA-32. J Surfactant Deterg. (2014) 17:669–82. doi: 10.1007/s11743-013-1519-4

[151] Nanthavanan P Arungandhi K Sunmathi D Niranjana J. Biological synthesis of keratin nanoparticles from dove feather (*Columba livia*) and its applications. Asian J Pharm Clin Res. (2019) 12:142–6. doi: 10.22159/ajpcr.2019.v12i10.34572

[152] Poole AJ Church JS Huson MG. Environmentally sustainable fibers from regenerated protein. Biomacromolecules. (2009) 10:1–8. doi: 10.1021/bm8010648 19035767

[153] Ramakrishnan N Sharma S Gupta A Alashwal BY. Keratin based bioplastic film from chicken feathers and its characterization. Int J Biol Macromol. (2018) 111:352–8. doi: 10.1016/j.ijbiomac.2018.01.037 29320725

[154] Reddy N Chen L Zhang Y Yang Y. Reducing environmental pollution of the textile industry using keratin as alternative sizing agent to poly (vinyl alcohol). J Clean Prod. (2014) 65:561–7. doi: 10.1016/j.jclepro.2013.09.046

[155] Reddy N Shi Z Temme L Xu H Xu L Hou X. Development and characterization of thermoplastic films from sorghum distillers dried grains grafted with various methacrylates. J Agric Food Chem. (2014) 62:2406–11. doi: 10.1021/jf405499t, PMID: 24601524

[156] Reddy N Yang Y. Structure and properties of chicken feather barbs as natural protein fibers. J Polym Environ. (2007) 15:81–7. doi: 10.1007/s10924-007-0054-7

[157] Rouse JG Van Dyke ME. A review of keratin-based biomaterials for biomedical applications. Dent Mater. (2010) 3:999–1014. doi: 10.3390/ma3020999

[158] Sharma S Gupta A Chik SMS Kee CG Mistry BM Kim DH. Characterization of keratin microparticles from feather biomass with potent antioxidant and anticancer activities. Int J Biol Macromol. (2017) 104:189–96. doi: 10.1016/j.ijbiomac.2017.06.015, PMID: 28596005

[159] Sharma S Gupta A SMSBT C CYG K Poddar PK. Dissolution and characterization of biofunctional keratin particles extracted from chicken feathers In: Proceedings of the IOP conference series: Materials science and engineering: IOP Publishing (2017)

[160] Tesfaye T Sithole B Ramjugernath D Chunilall V. Valorisation of chicken feathers: application in paper production. J Clean Prod. (2017) 164:1324–31. doi: 10.1016/j.jclepro.2017.07.034

[161] Tsuda Y Nomura Y. Properties of alkaline-hydrolyzed waterfowl feather keratin. Anim Sci J. (2014) 85:180–5. doi: 10.1111/asj.12093, PMID: 23865627

[162] Wang J Hao S Luo T Cheng Z Li W Gao F. Feather keratin hydrogel for wound repair: preparation, healing effect and biocompatibility evaluation. Colloids Surf B: Biointerfaces. (2017) 149:341–50. doi: 10.1016/j.colsurfb.2016.10.038, PMID: 27792983

[163] Xu H Cai S Xu L Yang Y. Water-stable three-dimensional ultrafine fibrous scaffolds from keratin for cartilage tissue engineering. Langmuir. (2014) 30:8461–70. doi: 10.1021/la500768b, PMID: 25010870

[164] Zhan M Wool RP. Mechanical properties of chicken feather fibers. Polymer Comp. (2011) 32:937–44. doi: 10.1002/pc.21112

[165] Hermosín I Chicón RM Dolores Cabezudo M. Free amino acid composition and botanical origin of honey. Food Chem. (2003) 83:263–8. doi: 10.1016/S0308-8146(03)00089-X

[166] Iglesias MT Martín-Álvarez PJ Polo MC de Lorenzo C González M Pueyo E. Changes in the free amino acid contents of honeys during storage at ambient temperature. J Agric Food Chem. (2006) 54:9099–104. doi: 10.1021/jf061712x, PMID: 17117796

[167] Alqarni AS Owayss AA Mahmoud AA Hannan MA. Mineral content and physical properties of local and imported honeys in Saudi Arabia. J Saudi Chem Soc. (2014) 18:618–25. doi: 10.1016/j.jscs.2012.11.009

[168] Andersen OM Markham KR. Flavonoids: Chemistry, biochemistry and applications. US: CRC press (2005).

[169] Castro-Vázquez L Díaz-Maroto MC Pérez-Coello MS. Aroma composition and new chemical markers of Spanish citrus honeys. Food Chem. (2007) 103:601–6. doi: 10.1016/j.foodchem.2006.08.031

[170] Kamal MA Klein P. Determination of sugars in honey by liquid chromatography. Saudi J biol sci. (2011) 18:17–21. doi: 10.1016/j.sjbs.2010.09.003, PMID: 23961099 PMC3730891

[171] Bonté F Desmoulière A. Le miel: origine et composition. Actual Pharmacol. (2013) 52:18–21. doi: 10.1016/j.actpha.2013.10.004

[172] Moreira RFA Maria CAB Pietroluongo M Trugo LC. Chemical changes in the non-volatile fraction of Brazilian honeys during storage under tropical conditions. Food Chem. (2007) 104:1236–41. doi: 10.1016/j.foodchem.2007.01.055

[173] Sak-Bosnar M Sakač N. Direct potentiometric determination of diastase activity in honey. Food Chem. (2012) 135:827–31. doi: 10.1016/j.foodchem.2012.05.006, PMID: 22868165

[174] Won S-R Li C-Y Kim J-W Rhee H-I. Immunological characterization of honey major protein and its application. Food Chem. (2009) 113:1334–8. doi: 10.1016/j.foodchem.2008.08.082

[175] da Silva PM Gauche C Gonzaga LV Costa ACO Fett R. Honey: chemical composition, stability and authenticity. Food Chem. (2016) 196:309–23. doi: 10.1016/j.foodchem.2015.09.051, PMID: 26593496

[176] Abbasi AM Khan MA Ahmad M Zafar M. Medicinal plant biodiversity of lesser. Himalayas-Pakistan: Springer Science & Business Media (2011).

[177] Chinlampianga M Singh RK Shukla AC. Ethnozoological diversity of Northeast India: empirical learning with traditional knowledge holders of Mizoram and Arunachal Pradesh. Indian J Tradit Knowl. (2013) 12:18–30.

[178] Deb AK Haque CE. ‘Every mother is a mini-doctor’: ethnomedicinal uses of fish, shellfish and some other aquatic animals in Bangladesh. J Ethnopharmacol. (2011) 134:259–67. doi: 10.1016/j.jep.2010.12.001 21185366

[179] Dixit A Kadavul K Rajalakshmi S Shekhawat M. Ethno-medico-biological studies of South India. Indian J Tradit Knowl. (2010) 9:116–8.

[180] Erejuwa OO Sulaiman SA Ab Wahab MS. Honey-a novel antidiabetic agent. Int J Biol Sci. (2012) 8:913–34. doi: 10.7150/ijbs.3697, PMID: 22811614 PMC3399220

[181] Jaroli D Mahawar MM Vyas N. An ethnozoological study in the adjoining areas of Mount Abu wildlife sanctuary. J Ethnobiol Ethnomed. (2010) 6:6. doi: 10.1186/1746-4269-6-6, PMID: 20144243 PMC2836285

[182] Mahawar MM Jaroli D. Animals and their products utilized as medicines by the inhabitants surrounding the Ranthambhore National Park. India J ethnobiol ethnomed. (2006) 2:46. doi: 10.1186/1746-4269-2-46, PMID: 17081314 PMC1636027

[183] Waykar B Alqadhi YA. Biological properties and uses of honey: A concise scientific review. Indian J Pharmaceutical Biolog Res. (2016) 4:20–7. doi: 10.30750/ijpbr.4.3.4

[184] Al-Waili N Salom K Al-Ghamdi A Ansari MJ Al-Waili A Al-Waili T. Honey and cardiovascular risk factors, in normal individuals and in patients with diabetes mellitus or dyslipidemia. J Med Food. (2013) 16:1063–78. doi: 10.1089/jmf.2012.0285 24328699

[185] Aluko EO Olubobokun TH Atang DE Nna VU. Honey’s ability to reduce blood pressure and heart rate in healthy male subjects. Front Sci. (2014) 4:8–11.

[186] Bobiş O Dezmirean DS Moise AR. Honey and diabetes: the importance of natural simple sugars in diet for preventing and treating different type of diabetes. Oxidative Med Cell Longev. (2018) 2018:1–12. doi: 10.1155/2018/4757893, PMID: 29507651 PMC5817209

[187] Cernak M Majtanova N Cernak A Majtan J. Honey prophylaxis reduces the risk of endophthalmitis during perioperative period of eye surgery. Phytother Res. (2012) 26:613–6. doi: 10.1002/ptr.3606, PMID: 22508360

[188] El-haskoury R Al-Waili N Kamoun Z Makni M Al-Waili H Lyoussi B. Antioxidant activity and protective effect of carob honey in CCl4-induced kidney and liver injury. Arch Invest Med. (2018) 49:306–13. doi: 10.1016/j.arcmed.2018.09.011, PMID: 30342848

[189] Erejuwa OO. Effect of honey in diabetes mellitus: matters arising. J Diabetes Metab Disord. (2014) 13:23. doi: 10.1186/2251-6581-13-23, PMID: 24476150 PMC3909917

[190] Francis A Cho Y Johnson DW. Honey in the prevention and treatment of infection in the CKD population: a narrative review. Evid Based Complement Alternat Med. (2015) 2015:1–8. doi: 10.1155/2015/261425 PMC4488250 26167189

[191] Li X Huang Q Ong C-N Yang X-F Shen H-M. Chrysin sensitizes tumor necrosis factor-α-induced apoptosis in human tumor cells via suppression of nuclear factor-kappaB. Cancer. (2010) 293:109–16. doi: 10.1016/j.canlet.2010.01.002 20133051

[192] Mandal MD Mandal S. Honey: its medicinal property and antibacterial activity. Asian Pac J Trop Biomed. (2011) 1:154–60. doi: 10.1016/S2221-1691(11)60016-6, PMID: 23569748 PMC3609166

[193] Omotayo EO Gurtu S Sulaiman SA Wahab MSA Sirajudeen K Salleh MSM. Hypoglycemic and antioxidant effects of honey supplementation in streptozotocin-induced diabetic rats. Int J Vitam Nutr Res. (2010) 80:74–82. doi: 10.1024/0300-9831/a000008, PMID: 20533247

[194] Oršolić N. Bee honey and cancer. J apiproduct and apimedical Sci. (2009) 1:93–103. doi: 10.3896/IBRA.4.01.4.01

[195] Rhone M Basu A. Phytochemicals and age-related eye diseases. Nutrition reviews.66:465-472. (2008) 66:465–72. doi: 10.1111/j.1753-4887.2008.00078.x 18667008

[196] Salehi A Jabarzare S Neurmohamadi M Kheiri S Rafieian-Kopaei M. A double blind clinical trial on the efficacy of honey drop in vernal keratoconjunctivitis. Evid Based Complement Alternat Med. (2014) 2014:1–4. doi: 10.1155/2014/287540 PMC3953621 24707307

[197] Vallianou NG Evangelopoulos A Skourtis A Kazazis C. Honey and cancer--A review. Current Topics in Nutraceutical Res. (2014) 12

[198] Vit P Jacob TJ. Putative anticataract properties of honey studied by the action of flavonoids on a lens culture model. J Health Sci. (2008) 54:196–202. doi: 10.1248/jhs.54.196

[199] Zhao H Cheng N He L Peng G Liu Q Ma T. Hepatoprotective effects of the honey of *Apis cerana* Fabricius on bromobenzene-induced liver damage in mice. J Food Sci. (2018) 83:509–16. doi: 10.1111/1750-3841.14021 29337369

[200] Umair M Altaf M Bussmann RW Abbasi AM. Ethnomedicinal uses of the local flora in Chenab riverine area, Punjab province Pakistan. J Ethnobiol Ethnomed. (2019) 15:7. doi: 10.1186/s13002-019-0285-4, PMID: 30709360 PMC6359778

[201] Faiz M Altaf M Umair M Almarry KS Elbadawi YB Abbasi AM. Traditional uses of animals in the Himalayan region of Azad Jammu and Kashmir. Front Pharmacol. (1951) 13. doi: 10.3389/fphar.2022.807831 PMC9277021 35847043

[202] Majeed M Bhatti KH Amjad MS Abbasi AM Bussmann RW Nawaz F. Ethno-veterinary uses of Poaceae in Punjab. Pakistan PloS one. (2020) 15:e0241705. doi: 10.1371/journal.pone.0241705, PMID: 33142315 PMC7608896

